# Neferine induces autophagy-dependent cell death in apoptosis-resistant cancers *via* ryanodine receptor and Ca^2+^-dependent mechanism

**DOI:** 10.1038/s41598-019-56675-6

**Published:** 2019-12-27

**Authors:** Betty Yuen Kwan Law, Francesco Michelangeli, Yuan Qing Qu, Su-Wei Xu, Yu Han, Simon Wing Fai Mok, Ivo Ricardo de Seabra Rodrigues Dias, Masood-ul-Hassan Javed, Wai-Kit Chan, Wei-Wei Xue, Xiao-Jun Yao, Wu Zeng, Hui Zhang, Jing-Rong Wang, Liang Liu, Vincent Kam Wai Wong

**Affiliations:** 1 State Key Laboratory of Quality Research in Chinese Medicine, Macau University of Science and Technology, Macau, China; 2 School of Biosciences, University of Birmingham, Edgbaston, Birmingham UK; 3 Department of Biological Sciences, University of Chester, Chester, UK; 4 Department of Basic Medicine of Zhuhai health school, Zhuhai, China; 5 College of Medicine, King Saud Bin Abdulaziz University of Health Sciences, King Abdul Aziz Medical City, Jeddah 21423 Kingdom of Saudi Arabia; 6 College of Chemistry and Chemical Engineering, Lanzhou University, Lanzhou, Gansu 730000 P.R. China

**Keywords:** Mitophagy, Mitophagy

## Abstract

Resistance of cancer cells to chemotherapy is a significant clinical concern and mechanisms regulating cell death in cancer therapy, including apoptosis, autophagy or necrosis, have been extensively investigated over the last decade. Accordingly, the identification of medicinal compounds against chemoresistant cancer cells *via* new mechanism of action is highly desired. Autophagy is important in inducing cell death or survival in cancer therapy. Recently, novel autophagy activators isolated from natural products were shown to induce autophagic cell death in apoptosis-resistant cancer cells in a calcium-dependent manner. Therefore, enhancement of autophagy may serve as additional therapeutic strategy against these resistant cancers. By computational docking analysis, biochemical assays, and advanced live-cell imaging, we identified that neferine, a natural alkaloid from *Nelumbo nucifera*, induces autophagy by activating the ryanodine receptor and calcium release. With well-known apoptotic agents, such as staurosporine, taxol, doxorubicin, cisplatin and etoposide, utilized as controls, neferine was shown to induce autophagic cell death in a panel of cancer cells, including apoptosis-defective and -resistant cancer cells or isogenic cancer cells, *via* calcium mobilization through the activation of ryanodine receptor and Ulk-1-PERK and AMPK-mTOR signaling cascades. Taken together, this study provides insights into the cytotoxic mechanism of neferine-induced autophagy through ryanodine receptor activation in resistant cancers.

## Introduction

Ryanodine receptors (RyRs) are located on the endoplasmic and sarcoplasmic reticulum (ER and SR) of most cells and are involved in intracellular calcium (Ca^2+^) release, representing an inevitable molecular component for the regulation of different cellular physiologies^1^. A direct correlation was found between RyR level and tumor grade^2^, for example, 4-chloro-m-cresol and caffeine, agonists of RyRs, could mobilize Ca^2+^ and suppress cancer growth^3,4^. On the other hand, intracellular Ca^2+^ signaling has been recognized as an important autophagy regulator^5^. Autophagy is a conserved self-degradation process for clearance and recycling of unwanted cellular materials, which is critical to the maintenance of cellular homeostasis. Owing to the important role of the autophagy machinery in the removal of damaged or long-lived cytosolic proteins and organelles, there are also strong correlations between autophagy defects and different pathological conditions, including tumorigenesis^6–8^.

Uncontrolled autophagy upregulation might lead to autophagic cell death in cancer cells. Consistently, loss of essential autophagy genes, such as Beclin 1, are found in different types of cancers, suggesting the pivotal role of autophagy in tumor suppression^6,9,10^. The benefit of several approved or experimental antitumor autophagic agents in cancer therapy, such as rapamycin, has been demonstrated through direct or indirect promotion of autophagic cell death^6,8–13^. Since resistance of cancer cells towards apoptosis is often developed after long-term chemotherapy, the induction of autophagic cytotoxicity in apoptosis-resistant tumor cells implies an alternative way for tumor suppression^6,9^. However, autophagy may also enable tumor cells survival by inducing stress tolerance in response to chemotherapy^7^. In addition, type 3 RyR (RyR3) has been reported to participate in autophagy-induced cell death in neural stem cells^4^. Together with the fact that RyRs are important to the manipulation of cytosolic Ca^2+^ levels, it is tempting to question if the autophagy-dependent cell death observed in cancers, or more specifically apoptosis-resistant cancers, are related to the Ca^2+^ regulatory function of RyRs as well. As such, elucidating the complex roles and mechanisms of autophagy in tumorigenesis is crucial to the identification of novel therapeutic agents.

Neferine, an alkaloid isolated from *Nelumbo nucifera*, has the capacity of limiting cancer growth^14–17^. Recent findings have suggested that neferine is able to induce autophagy in cancer and neuronal cells^16,18^. Thus far, the precise mechanisms associated with the neferine-induced autophagic effect in cancer models (especially resistant cancers) has remained elusive. This study reports for the first time that neferine activates the RyR, leading to Ca^2+^ release and autophagy induction *via* the ULK/CaMKK- AMP-activated protein kinase (AMPK)-mammalian target of rapamycin (mTOR)-dependent pathway. Besides, neferine induces cytotoxicity in a panel of apoptosis-resistant cell lines *via* autophagic cell death. The newly identified RyR-mediated autophagic mechanism of neferine suggests the clinical relevance towards apoptosis-resistant cancers providing insights into the exploitation of novel interventions.

## Results

### Neferine induces cytotoxicity and GFP- light-chain 3 (LC3) puncta formation in various cancer cell lines

**Figure 1:**
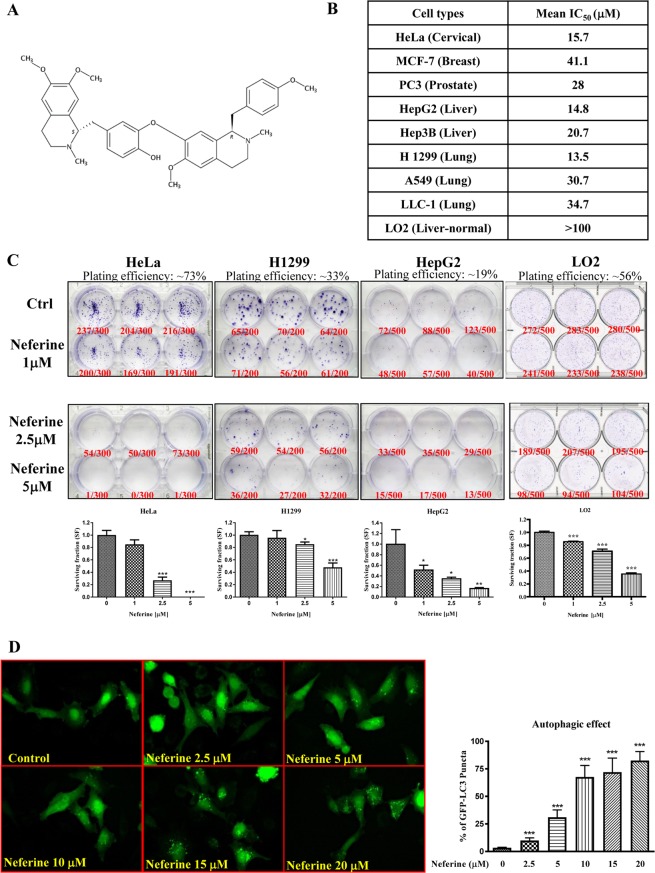
Neferine dose-dependently suppresses cancer cells growth and activates autophagy induction. (**A**) Chemical structure of Neferine. (**B**) Cytotoxicity (IC_50_) of neferine towards different types of cancer and the control LO2 cell line. The MTT graphs are presented in Supplementary Fig. S1. (**C**) Bright field images showing the colony formation of HeLa, H1299, and HepG2 cancer cells in response to neferine treatments (1 μM, 2.5 μM and 5 μM) for 14 days. Plating efficiency (PE) = no. of colonies formed/ no. of cells seeded x 100%; surviving fraction (SF) = no. of colonies formed after treatment/ no. of cells seeded x PE. Bar chart represents the quantitation of SF upon the neferine treatment. (**D**) EGFP-LC3 detection of neferine-mediated autophagy in HeLa cells. HeLa cells were transiently transfected with the EGFP-LC3 plasmid for 24 h and then treated with DMSO (Control), or indicated concentrations of neferine for 4 h. Representative micrographs of cells that show EGFP-LC3 localization. Bar chart represents the quantitation of autophagic cells. Percentages of autophagic cells demonstrated by the increased number of cells with EGFP-LC3 dots signal (≥10 dots/cell) over the total number of EGFP-positive cells in the same field. More than 1000 EGFP-positive cells were scored for each treatment. Data are the means of three independent experiments; error bars, S.D. ***P < 0.001 for neferine treated cells. Images shown are representative of three independent experiments. All images are captured under 60X objective magnification.

We firstly demonstrated that neferine, isolated from *Nelumbo nucifera* (Fig. 1A), induced cell death in a panel of cancer and apoptosis-resistant cancer cells. Different cancer cells, including HeLa, MCF-7, PC3, HepG2, Hep3B, H1299, A549 and LLC-1, were used for cell cytotoxicity assay with normal human hepatocytes LO2 served as control. In Fig. 1B and Supplementary Fig. S1, neferine is shown as less toxic in MCF-7 breast cancer cells (mean IC_50_ = 41.1 μM), A549 lung cancer cells (mean IC_50_ = 30.7 μM), and LLC-1 lung cancer cells (mean IC_50_ = 34.7 μM), but potently cytotoxic to HeLa, HepG2, and H1299 cancer cells (mean IC_50_ = 13.5–15.7 μM). The cytotoxicity of neferine was the lowest in LO2 (mean IC_50_ > 100 μM), suggesting that the neferine cytotoxic effects was relative cancer cell specific. *In vitro* clonogenic cell survival assay was used to determine the effectiveness of neferine by using the most sensitive cancer cells (i.e. HeLa, H1299, and HepG2 cells) and LO2 normal hepatocytes. All tested cancer cell colonies were significantly reduced upon 5 μM neferine exposure, confirming the potential anti-cancer property of neferine, whereas LO2 cell colonies reduced slightly upon 1, 2.5, and 5 μM neferine exposures compared to cancer cells (Fig. 1C), suggesting the cancer cell-specific property of neferine in anti-colony-formation. As shown by the increased number of HeLa cells containing GFP-LC3 puncta (autophagy marker) (Fig. 1D), neferine exhibits a dose-dependent increase in autophagy induction.

**Figure 2:**
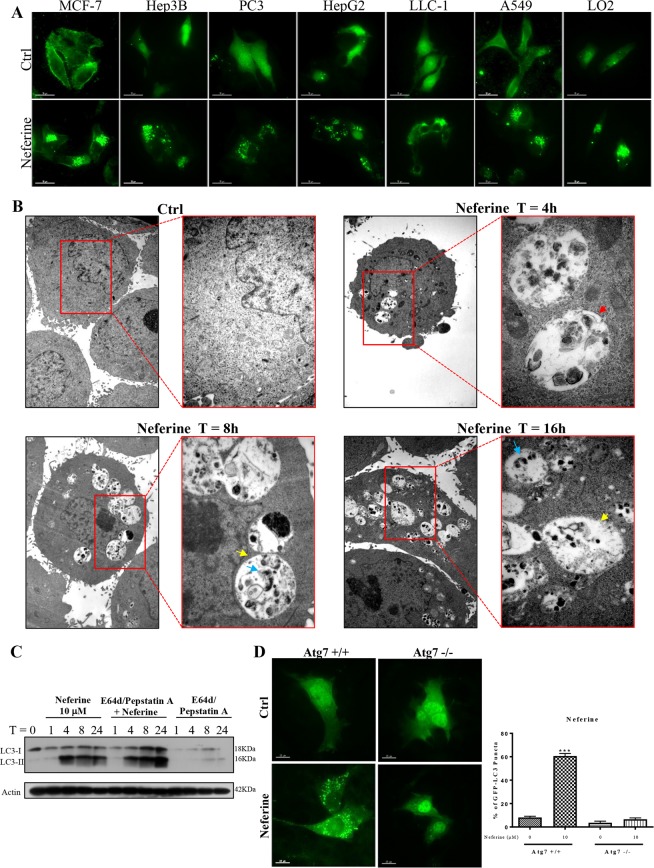
Neferine-induced autophagy and LC3-II conversion depend on autophagic gene, Atg7. (**A**) EGFP-LC3 puncta detection of neferine-mediated autophagy in other cancer and normal cells. Cancer cells (MCF-7, Hep3B, PC3, HepG2, LLC-1, and A549) and normal liver hepatocytes (LO2) were transiently transfected with the EGFP-LC3 plasmid for 24 h and then treated with DMSO (Ctrl), or 10 μM of neferine for 4 h. The quantification graph is presented in Supplementary Fig. S2. (**B**) Neferine induced the formation of autophagosomes and autolysosomes. Representative electron micrographs showing the ultra-structures of HeLa cells treated with neferine (10 μM) at indicated times. All images were captured with 40000X magnification; micrographs from red rectangle represent the magnified cropped images of the double-membrane autophagosomes (red arrow), autolysosomes (yellow arrow) and engulfed organelles (blue arrow). (**C**) Neferine induced autophagic flux in HeLa cells. Cells were treated with neferine (10 μM) in the presence or absence of 10 μg/mL lysosomal protease inhibitors (E64d and pepstatin A) for 24 h. Cell lysates were analyzed by western blot for LC3-II conversion (LC3-I, 18 kDa; LC3-II, 16 kDa), and actin respectively. The full-length blots/gels are presented in Supplementary Fig. S9. (**D**) Neferine-mediated autophagy depended on Atg7 expression. Both Atg7^+/+^ wild-type and Atg7^−/−^ deficient MEFs were transiently transfected with the EGFP-LC3 plasmid for 24 h and then treated with DMSO (Ctrl) or 10 μM neferine for 4 h. The cells were then fixed for fluorescence imaging and cells counting. Bar chart represents the quantification of autophagic cells. Percentages of autophagic cells demonstrated by the increased number of cells with EGFP-LC3 dots signal (≥10 dots/cell) over the total number of EGFP-positive cells in the same field. More than 1000 EGFP-positive cells were scored for each treatment. Data are the means of three independent experiments; scale bar, 15 μm; error bars, S.D. ***P < 0.001 for Atg7^+/+^ MEFs treated with or without Nef.

In addition, Fig. 2A and Supplementary Fig. S2 showed that 10 μM of neferine significantly induced GFP-LC3 puncta formation in all the assayed cancer cells and control, indicating the non-cell type-specific nature of the induced autophagic effect. The ultrastructure of neferine-treated HeLa cells was analyzed by transmission electron microscopy. Numerous double-membraned autophagosomes were observed in a dose-dependent manner upon neferine treatment (10 μM) together with the autolysosomes containing engulfed organelles (Fig. 2B). For the purpose of monitoring the autophagic flux, we measured LC3-II formation by western blot in the presence of lysosomal protease inhibitors (pepstatin A and E64d)^6^. As expected, neferine significantly increased the rate of LC3-II formation in the presence of the inhibitors when compared with using either inhibitors or neferine alone (Fig. 2C). Therefore, neferine-induced autophagic activity was a result of enhanced autophagosome formation.

### Neferine depends on Atg7 for autophagy induction

Autophagy-related gene 7 (Atg7) is necessary for triggering autophagy and the corresponding cell death^6,9,10,19–21^. To clarify if Atg7 is critical to neferine-induced autophagy, we incubated GFP-LC3-transfected wild-type and Atg7-deficient (Atg7^−/−^) mouse embryonic fibroblasts (MEFs) with neferine for 24 h and subjected them to GFP-LC3 puncta quantification. Neferine significantly induced GFP-LC3 puncta formation in wild-type MEFs, but not in the Atg7^−/−^ counterpart (Fig. 2D), indicating Atg7 involvement in neferine-mediated autophagy induction.

### Up-regulated ULK-1 and pancreatic ER kinase (PERK) gene expression in neferine-induced autophagy

**Figure 3:**
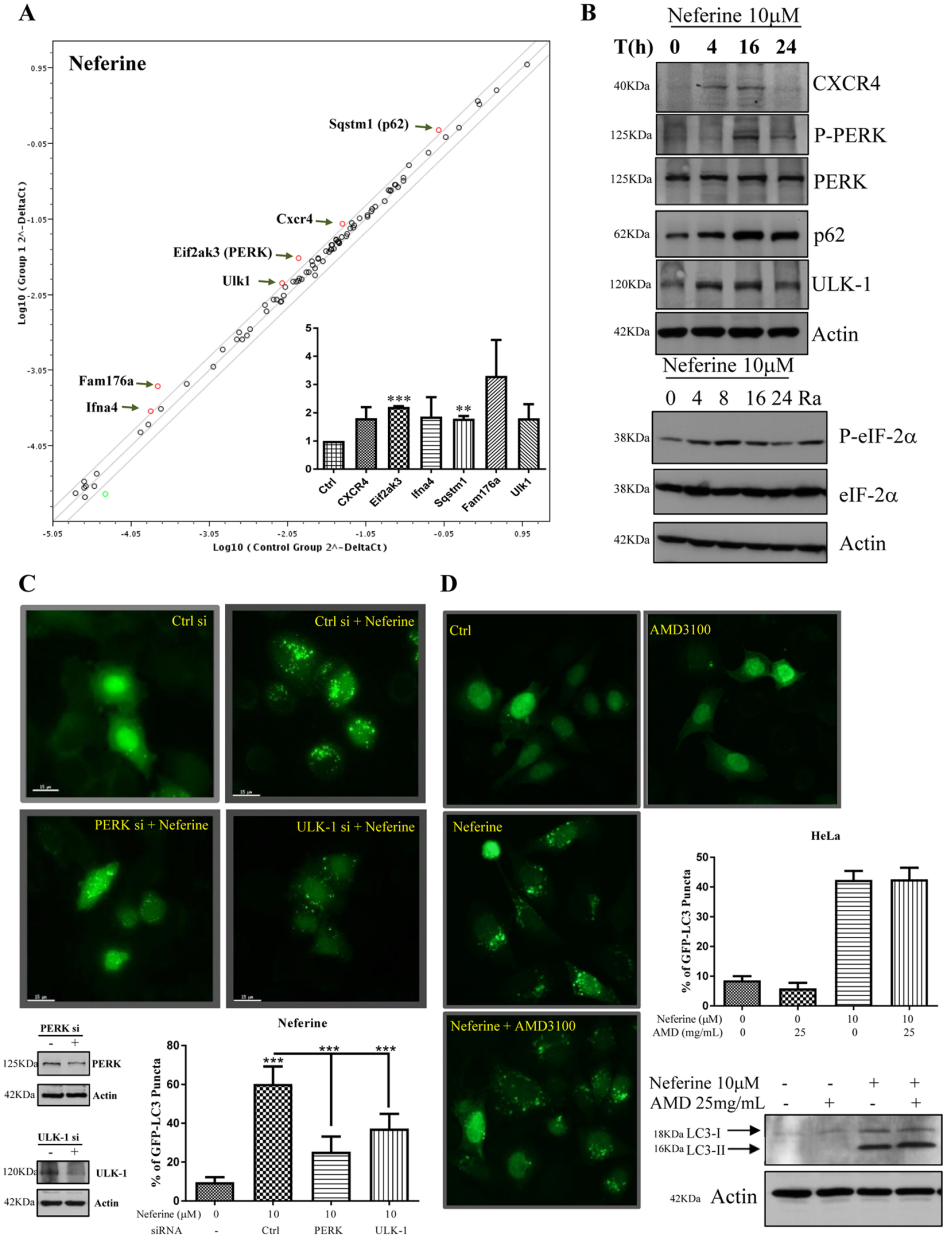
Gene regulation of neferine-mediated autophagy induction. (**A**) RT^2^ profiler autophagy PCR array analysis of neferine. HeLa cells were with treated with 10 μM of neferine for 24 h. The total RNA was extracted and reverse-transcripted as cDNA. Real-time PCR reactions were performed using the RT2 SYBR® Green qPCR Mastermix and data analysis was determined using the Qiagen’s integrated web-based software package for the PCR Array System. Scatter plot highlighted the up-regulation and down-regulation of genes in response to neferine treatment. Inner panel: quantification of PCR array analysis. (**B**) Neferine-mediated genes regulation was confirmed by western blotting. Upper panel, HeLa cells were treated with neferine (10 μM) for the indicated time. Cell lysates were analyzed with antibodies against CXCR4, P-PERK, PERK, p62, ULK-1 and actin respectively. Lower panel, HeLa cells were treated with neferine (10 μM) for the indicated time and rapamycin (300 nM) for 24 h. Cell lysates were analyzed with antibodies against P-eIF-2α, eIF-2α and actin respectively. The quantification graphs and full-length blots/gels are presented in Supplementary Figs. S2 and S10 (**A**), respectively. (**C**) Activation of PERK and ULK-1 is required for neferine-induced autophagy. HeLa cells were transfected with control *si*RNA, PERK or ULK-1 *si*RNA together with EGFP-LC3 plasmid for 48 h, cells were treated with neferine (10 μM) for 4 h and then fixed for fluorescence imaging and cells counting. Western blot images indicate the gene knock down efficiency. Bar chart represents the quantitation of autophagic cells. The full-length blots/gels are presented in Supplementary Fig. S10 (**B**). (**D**) Effect of CXCR4 in Nef-induced autophagy. EGFP-LC3 transfected HeLa cells were treated with 10 μM neferine in the presence or absence of CXCR4 specific inhibitor, AMD 3100 (25 mg/mL) for 4 h. The cells were then fixed for fluorescence imaging and cells counting. Bar chart represents the quantitation of autophagic cells. Western blot image indicates the LC3-II conversion in HeLa cells in response to neferine and AMD 3100 treatment. The full-length blots/gels are presented in Supplementary Fig. S10 (**C**). Percentages of autophagic cells demonstrated by the increased number of cells with EGFP-LC3 dots signal (≥10 dots/cell) over the total number of EGFP-positive cells in the same field. More than 1000 EGFP-positive cells were scored for each treatment. Error bars, S.D. **P < 0.01, ***P < 0.001 for neferine-treated HeLa cells with or without PERK/ULK-1 *si*RNA knockdown.

RT² Profiler™ PCR array analysis targeting 87 autophagic-related genes showed that neferine induced autophagy through regulation of a panel of genes, including Ifna4, Fam176a, Ulk-1, PERK, Cxcr4, and p62 (SQSTM1) (Fig. 3A). Among these genes, the up-regulation of Cxcr4, p-PERK, PERK, SQSTM1, and Ulk-1 were validated by Western blot analysis (Fig. 3B and Supplementary Fig. S2). Consistently, the downstream target of PERK, eIF2-α^22^, was activated by phosphorylation (Fig. 3B, lower panel and Supplementary Fig. S2). Ra (rapamycin) that induced autophagy by inhibiting mTOR activity was used as positive control to demonstrate the effect of autophagy on the phosphorylation of eIF-2. SQSTM1 is a well-known autophagic substrate for monitoring autophagic flux as indicated by the auto-lysosomal degradation of p62^23^. Since neferine-induced autophagy was associated with p62 up-regulation (Fig. 3B), real-time PCR analysis was performed and we confirmed that p62 mRNA level was significantly up-regulated after neferine treatments (Fig. 3A, inner panel). Therefore, neferine may affect the transcriptional level of p62 which suggests special caution when using p62 as a marker to evaluate autophagy induction^6^.

In line with the PCR array results, siRNA knockdown of PERK or Ulk-1 genes abolished the neferine-mediated GFP-LC3 puncta formation, confirming the involvement of PERK^24^ and Ulk-1^25^ in neferine-mediated autophagy (Fig. 3C). Moreover, AMD3100, a Cxcr4 inhibitor^26^, failed to suppress neferine-mediated GFP-LC3 puncta formation and LC3-II conversion (Fig. 3D), suggesting that Cxcr4 gene is not involved in neferine-induced autophagy. Apparently, the autophagy-inducing effect of neferine is mediated by the Ulk-1 and PERK signaling pathways.

### AMPK-mTOR signaling cascade is activated during neferine-mediated autophagy-dependent cell death

**Figure 4:**
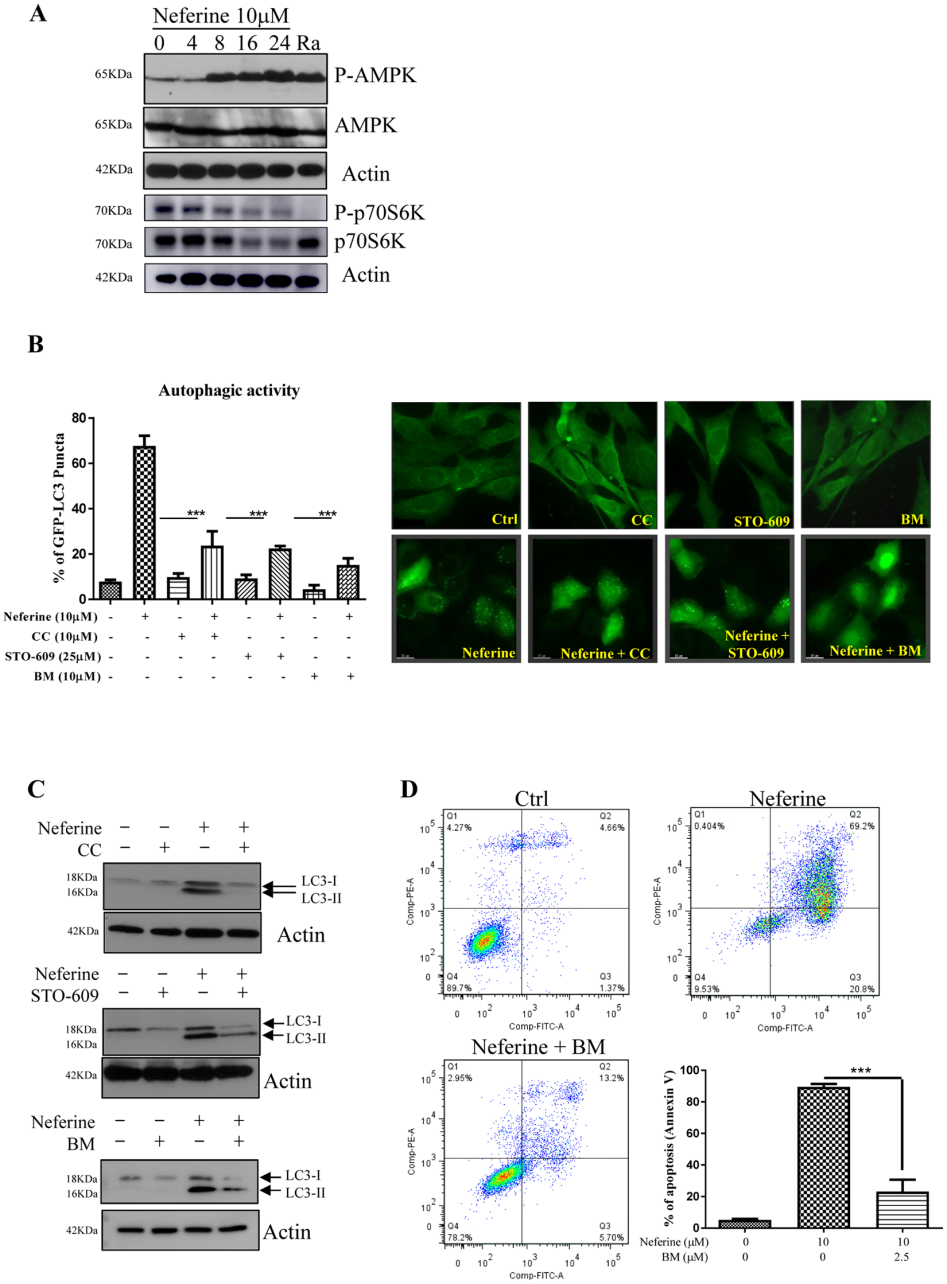
Role of CaMKKβ-AMPK-mTOR signaling cascade in neferine-mediated autophagy. (**A**) Neferine activated AMPK-mTOR signaling pathways. HeLa cells were treated with neferine (10 μM) for the indicated time and rapamycin, Ra (300 nM) for 24 h. The cells lysate was then analyzed for p-AMPK, AMPK, p-p70S6K, total p70S6K, and actin respectively. The quantification graphs and full-length blots/gels are presented in Supplementary Figs. S3 and S11 (**A**), respectively. (**B**) Inhibitors for AMPK, CaMKKβ and calcium chelator abrogated the neferine-mediated autophagic effect in HeLa cells. HeLa cells were transiently transfected with the EGFP-LC3 plasmid for 24 h and then treated with DMSO (Ctrl), or 10 μM neferine with or without 10 μM of AMPK inhibitor compound C (CC), 25 μM of CaMKKβ inhibitor STO-609 and 10 μM of calcium chelator BAPTA/AM (BM) for 4 h. The cells were then fixed for fluorescence imaging and cells counting. Bar chart represents the quantitation of autophagic cells. (**C**) Inhibitors for AMPK, CaMKKβ and calcium chelator suppressed the neferine-induced LC3-II conversion in HeLa cells. HeLa cells were treated with DMSO (Ctrl), or 10 μM neferine with or without 10 μM of AMPK inhibitor compound C (CC), 25 μM of CaMKKβ inhibitor STO-609 and 10 μM of calcium chelator BAPTA/AM (BM) for 24 h. Cell lysates were analyzed by western blot for LC3-II conversion (LC3-I, 18 kDa; LC3-II, 16 kDa), and actin respectively. The full-length blots/gels are presented in Supplementary Fig. S11 (**B**). (**D**) Calcium chelator, BM inhibits the neferine-induced cell death in HeLa cancer cells. HeLa cells were treated with DMSO (Ctrl), neferine with or without 2.5 μM of BAPTA/AM (BM) for 24 h. The treatment-induced cell death was then measured by flow cytometry analysis after annexin V staining. Bar chart represents the percentage of apoptosis. Data are the means of three independent experiments; error bars, S.D. ***P < 0.001; scale bar, 15 μm.

Since, AMPK is one of the important regulators of autophagy, we further investigated if neferine is able to activate the phosphorylation of AMPK^9^. Our results showed a time-dependent increase of AMPK phosphorylation after neferine treatment (Fig. 4A). The AMPK phosphorylation was followed by a reduction in phosphorylated p70S6K, the downstream mTOR target, after 4–8 hours of neferine treatment. Moreover, p70S6K phosphorylation was significantly increased after 16 hours (Fig. 4A & Supplementary Fig. S3), which may be due to re-occurrence of cellular p70S6k phosphorylation resulting from feedback activation of Akt/mTOR^27,28^. Consistently, a significant reduction in neferine-induced GFP-LC3 puncta was observed in cells pre-treated with the AMPK inhibitor CC (Fig. 4B), confirming the involvement of AMPK.

As the upstream signaling mediator of AMPK, CaMKK regulates the phosphorylation of AMPK into its active form^9^. In order to investigate the role of CaMKK in neferine-induced autophagy, HeLa cells were treated with neferine in the presence of the CaMKK inhibitor STO-609^29^. STO-609 significantly reduced the percentage of cells containing GFP-LC3 puncta (Fig. 4B), suggesting the possible role of Ca^2+^ signaling in the autophagic process. Concomitantly, addition of the intracellular Ca^2+^ chelator BM abolished GFP-LC3 puncta formation (Fig. 4B). As shown by Western blot, the neferine-mediated LC3-II conversion was also markedly suppressed by CC, STO-609, and BM (Fig. 4C and Supplementary Fig. S3). BM not only suppressed neferine-induced autophagy, but also rescued cell death in response to neferine treatment (Fig. 4D). Collectively, neferine induced autophagy and its dependent cell death *via* the Ca^2+^-mediated Ca^2+^/calmodulin-dependent kinase kinase-β (CaMKKβ)-AMPK-mTOR signaling pathway.

### Neferine directly targets RyR to increase cytosolic [Ca^2+^] for autophagy and cell death induction

**Figure 5:**
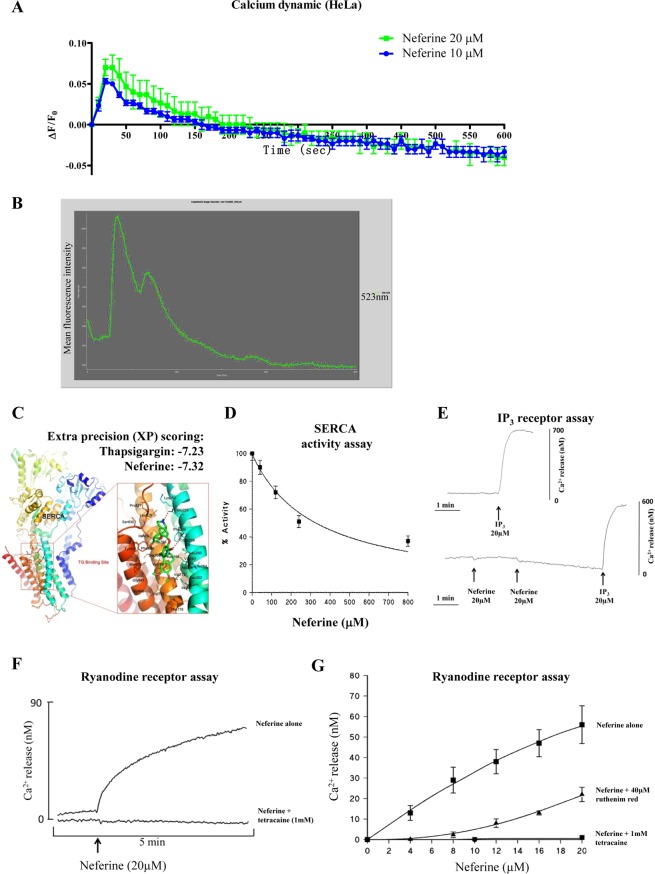
Neferine activates ryanodine receptor in turn to mobilize cytosolic calcium level. (**A**) Neferine induced calcium dynamic change in HeLa. HeLa cells stained with FLIPR Calcium 6 Assay Kit were treated with 10 and 20 μM of neferine, then immediately subjected to calcium dynamic measurement by FLIPR Tetra High-Throughput Cellular Screening System. Data from the chart represents mean values ± S.D. of three independent experiments. (**B**) Single-cell imaging of Neferine-mediated mobilization of cytosolic calcium. HeLa cells seeded in 35 mm confocal disc were incubated with 5 μM of Fluo 3/AM in HBSS buffer at 37 °C for 30 min. Cells were then washed with HEPES buffer saline and incubated at 37 °C for another 10 min. Calcium signal was monitored by Applied Precision DeltaVision Elite in real-time mode for consecutive 5 min during the addition of 10 μM neferine in HBSS buffer. Chart represents the mean intensity of fluorescence signal at 523 nm. (**C**) Computation docking of SERCA with neferine. The SERCA protein was represented as ribbon diagram. Key residues around the binding pocket are shown as lines and the neferine is presented as sticks. The hydrogen bonds were labeled as red dashed lines. Extra precision (XP) scoring for thapsigargin and neferine are −7.23 and −7.32 respectively. (**D**) Inhibition of Ca^2+^ ATPase (SERCA) activity in skeletal muscle SR by neferine. Experiments were measured at 25 °C (pH 7.2) using the coupled enzyme assay as described in^10^. (**E**) Ca^2+^ releasing effect of neferine on inositol 1,4,5-trisphosphate (InsP_3_) receptor. The InsP_3_ receptor rich rat brain microsomes were pre-incubated with or without neferine (20 or 40 μM), and then subjected to IP_3_ (20 μM) treatment. The calcium release was immediately measured within 5 min. (**F**) Time-dependent Ca^2+^ release from skeletal muscle SR upon addition of neferine. The ryanodine receptor rich skeletal muscle SR was pre-incubated with or without RyR inhibitor, tetracaine (1 mM) for 1 min and then subjected to neferine (20μM) treatment. The calcium release was immediately measured within 5 min. (**G**) Dose-dependent Ca^2+^ release from skeletal muscle SR upon addition of neferine. The ryanodine receptor rich skeletal muscle SR was treated with indicated concentrations of neferine in the presence or absence of RyR inhibitor, ruthenium red (40 μM) or tetracaine (1 mM). The calcium release was immediately measured by Fluo-3 (free acid). All experimental points are the mean ± SD of 3 determinations.

In order to confirm whether neferine mediates autophagy through increasing cytosolic [Ca^2+^] levels, FLIPR Calcium 6 assay was performed to demonstrate that neferine dose-dependently induced calcium dynamic change in HeLa cancer cells (Fig. 5A). Furthermore, single live-cell Ca^2+^ imaging was monitored and the results showed that HeLa cells loaded with Fluo 3-AM displayed strikingly increased fluorescence intensity upon 10 μM neferine treatment (Fig. 5B & Supplementary Video-1). Flow cytometry analysis demonstrated consistent results in which the neferine-treated HeLa cells loaded with Fluo 3 displayed a dose- and time-dependent increase in fluorescence signal, indicating a rise in intracellular Ca^2+^ levels (Supplementary Fig. S4).

Since sarcoplasmic/endoplasmic reticulum Ca^2+^ ATPase pump (SERCA) is responsible for transporting cytoplasmic Ca^2+^ into the sarcoendoplasmic reticulum and SERCA inhibition can induce autophagy^9,10^, neferine might also inhibit SERCA to induce autophagy. Computational ligand docking studies were performed to evaluate the possible neferine-SERCA interactions. Comparative analysis of the low-energy ligand conformations found the preferred site for neferine within the transmembrane domain (Fig. 5C), close to the binding site of TG, a potent inhibitor of the SERCA pump which induces autophagy through perturbing Ca^2+^ homeostasis^30^. Figure 5C illustrated the structures of neferine docked into the SERCA binding site of TG. In the predicted binding pose of neferine, the hydrophobic groups bound into the hydrophobic pocket, making favorable hydrophobic effects and van der Waals interactions with residues Phe256, Leu260, Val263, Leu266, Ile267, Ala270, Ala305, Ala306, Pro308, Ile756, Ile761, Val769, Val772, Val773, Phe776, Leu777, Pro827, Leu828, Ile829, Phe834, Met838, Gly841, and Gly842. Neferine made less hydrophobic and van der Waals interactions with SERCA, however, two hydrogen bonds were identified between neferine and residues Glu255 and Gln259. Comparison of the docking pose of neferine with TG (XP docking score: −7.32 vs. −7.23 kcal/mol) indicated that the two compounds were located within the SERCA binding pocket.

To ascertain whether SERCA was suppressed by neferine, we investigated its biological effect using purified rabbit skeletal muscle SR membranes expressing the SERCA1A isoform^31^. Results showed that the SERCA activity was inhibited by neferine in a dose-dependent manner (Fig. 5D), however, neferine is a very weak inhibitor of SERCA with an IC_50_ value of 320 ± 70 μM. In addition, we determined whether neferine mobilizes Ca^2+^ release *via* inositol 1,4,5-trisphosphate (InsP_3_) receptor, which is another SR Ca^2+^ channel expressed in many cell types, including brain microsomes^32^. As shown in Fig. 5E, top trace indicated Ca^2+^ released from rat brain microsomes upon IP_3_ treatment which was not illustrated in bottom trace (neither at 20 nor 40 μM). Whereas the IP_3_ response was unaffected by neferine as similar to top trace (control). RyR is another Ca^2+^-releasing channel expressed in a variety of cells, in particular, the skeletal muscle^32^. Figure 5F showed a robust Ca^2+^ release from neferine-treated skeletal muscle SR. However, 1 mM administration of tetracaine, a RyR inhibitor, completely blocked the neferine-induced Ca^2+^ release (Fig. 5F). Furthermore, the dose-dependent effect of neferine on Ca^2+^ release from SR was completely abolished in the presence of 1 mM tetracaine (Fig. 5G), whereas in the presence of 40 μM ruthenium red (another RyR inhibitor), the neferine-mediated Ca^2+^ release was substantially reduced, especially at low concentrations of neferine (Fig. 5G). Our results suggested that neferine mobilizes cytosolic Ca^2+^ specifically *via* RyR activation.

**Figure 6:**
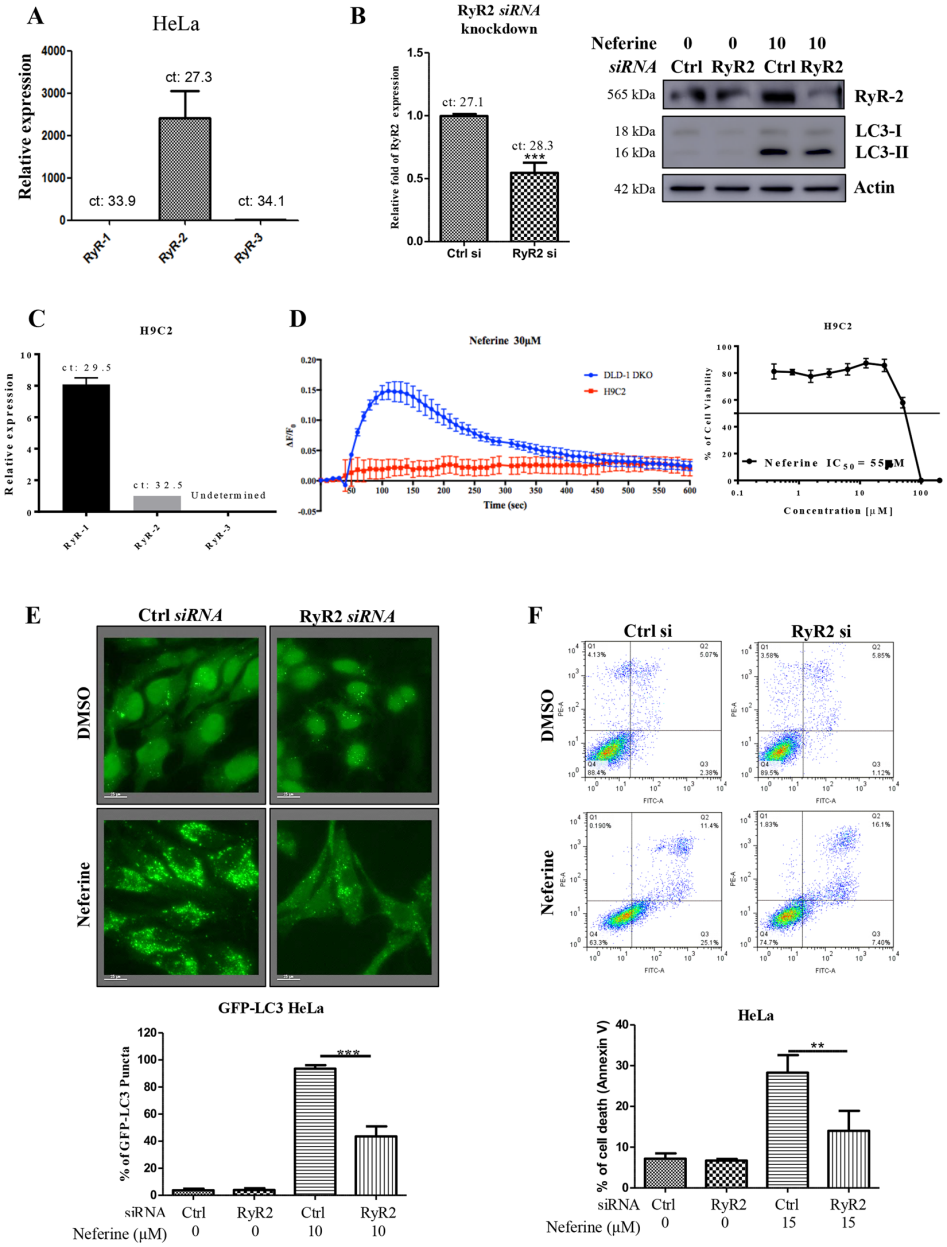
Neferine induces autophagy and cell death *via* ryanodine receptor (RyR) activation. (**A**) Gene expression profile of ryanodine receptors in HeLa cancer cells. (**B**) Knockdown of RyR2 gene abolished neferine-mediated LC3-II conversion. HeLa cancer cells were *siRNA* knockdown of RyR2, the cells were then treated with 10 μM of neferine for 24 h. Cell lysates were analyzed by Western blot for RyR2 (Santa Cruz: sc-13942; RyR2, 565 kDa) and LC3-II conversion (LC3-I, 18 kDa; LC3-II, 16 kDa), and actin respectively. The full-length blots/gels are presented in Supplementary Fig. S12. Bar chart represented the knockdown efficiency of RyR2 gene in HeLa cells. (**C**) Gene expression profile of ryanodine receptors in H9C2 rat cardiac myoblasts. (**D**) Neferine induced higher calcium dynamic response in cancer cells and demonstrated less cytotoxicity in the cardiac myoblasts. Both DLD-1 Bax-Bak DKO colon cancer cells and H9C2 rat myoblasts stained with FLIPR Calcium 6 Assay Kit were treated with 30 μM of neferine, then immediately subjected to calcium dynamic measurement by FLIPR Tetra High-Throughput Cellular Screening System. Data from the chart represents mean values ± S.D. Of three independent experiments. H9C2 rat myoblasts cells were treated with neferine from 0 to 100 μM for 72 h. Cell viability was then measured by cell cytotoxicity assay. (**E**) Knockdown of RyR2 gene abolished neferine-mediated autophagy induction. GFP-LC3 stable HeLa cells were *siRNA* knockdown of RyR2, the cells were then treated with 10 μM of neferine for 24 h. The cells were then fixed for fluorescence imaging and cells counting. Bar chart represents the quantification of autophagic cells. Percentages of autophagic cells demonstrated by the increased number of cells with GFP-LC3 dots signal (≥10 dots/cell) over the total number of GFP-positive cells in the same field. Data are the means of three independent experiments; scale bar, 15 μm; error bars, S.D. ***P < 0.001 for neferine-treated cells with or without RyR2 knockdown. (**F**) Knockdown of RyR2 gene reduced neferine-mediated cell death. HeLa cancer cells were *siRNA* knockdown of RyR2 gene, the cells were then treated with 15 μM of neferine for 24 h. The cell cytotoxicity was then assayed by annexin V staining using flow cytometry. Results shown are the means ± S.E.M. of three independent experiments.

To investigate the role of RyR on autophagy and cell death induction in cancer, we silenced the RyR in HeLa cancer cells by using specific *siRNA* and monitored the corresponding neferine-mediated autophagy and cell death. Real-time PCR results confirmed that the RyR isoform 2 (RyR2) was mainly expressed in HeLa cancer cells (Fig. 6A). However, except MCF-7, the cytotoxicity of neferine seems to be relatively correlated with the expression level of RyR1 in HeLa, HepG2, Hep3B and H1299 cell lines, whereas the expression of RyR2 may have an additive effect in neferine-mediated cytotoxicity in HeLa and Hep3B cells (Supplementary Fig. S5).

Notably, specific *siRNA* knockdown of RyR2 significantly down-regulated RyR2 protein expression, which concomitantly suppressed the neferine-mediated LC3-II conversion (Fig. 6B). To further determine whether neferine can affect heart tissue *via* the activation of RyR-mediated Ca^2+^ mobilization, real-time PCR of RyR isoforms on cardiac myoblasts was performed. We found that RyR isoform 1 (RyR1) was slightly expressed in H9C2 rat cardiac myoblasts (Fig. 6C). Concomitantly, calcium dynamic assay has revealed that H9C2 myoblasts were much less sensitive in Ca^2+^ response compared to DLD-1 colon cancer cells upon high dosage of neferine treatment (30μM) (Fig. 6D, left panel). These results were consistent with the observation that neferine was relatively less toxic to H9C2 myoblasts (IC_50_ = 55 μM) due to their relatively low expression level of RyR isoforms (Fig. 6D, right panel). In addition, neferine-mediated GFP-LC3 puncta formation and cell death were markedly diminished in HeLa cancer cells silenced with RyR2 *siRNA* (Fig. 6E,F). Taken together, RyR has an indispensable role in small-molecule-mediated autophagy and cell death.

### Neferine induces autophagy-dependent cell death in apoptosis-resistant cancer via RyR activation

**Figure 7:**
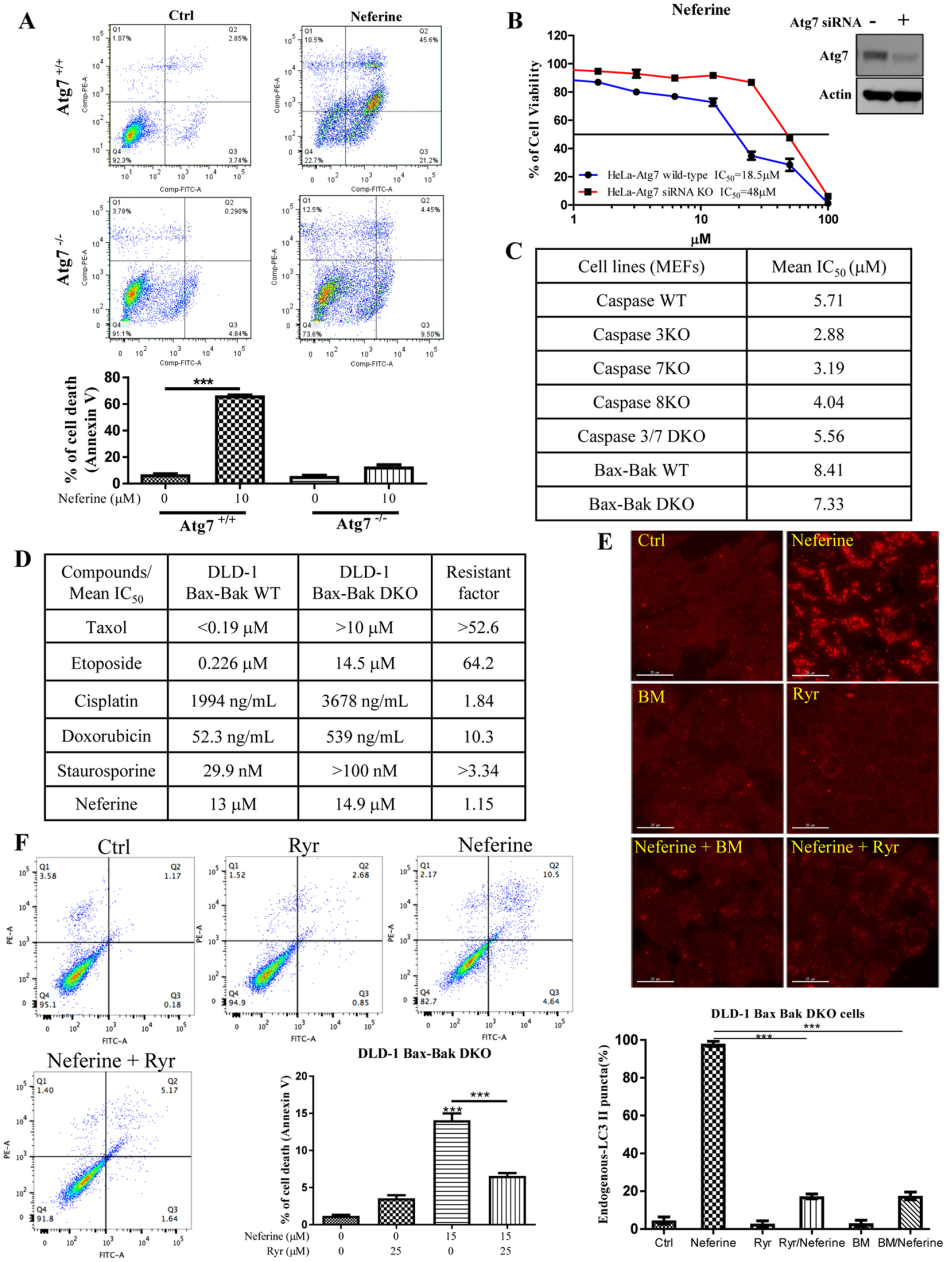
Neferine induces autophagic cell death in apoptosis-resistant cancer *via* ryanodine receptor activation. (**A**) Annexin V flow cytometry analysis of neferine on Atg7 wild-type and deficient MEFs. (**B**) HeLa cells transfected with Atg7 siRNA or non-targeting control siRNA for 24 h were treated with neferine from 0 to 100 μM for 72 h. Cell viability was then measured by cell cytotoxicity assay. Results shown are the means ± S.E.M. of three independent experiments. Western blot detection of Atg7 in HeLa cells transfected with control siRNA or Atg7 siRNA after 24 h. The full-length blots/gels are presented in Supplementary Fig. S13. (**C**) Cytotoxicity (IC_50_) test of neferine towards apoptosis-resistant cells with different caspase or Bax-Bak deficiency MEFs. (**D**) Cytotoxicity (IC_50_) of neferine and different chemotherapeutic agents towards the DLD-1 Bax-Bak WT and apoptotic-resistant DLD-1 Bax-Bak DKO cell lines and drug resistance analysis (resistant factor) of the two cell lines. (**E**) Calcium chelation or Ryanodine receptor inhibition abrogated the neferine-mediated autophagic effect in apoptosis-resistant cancer cells. DLD-1 Bax-Bak DKO colon cancer cells were treated with DMSO (Ctrl), or 10 μM neferine with or without 10 μM of the calcium chelator BAPTA/AM (BM), or with 25 μM of Ryanodine receptor inhibitor, ryanodine (Ryr) for 4 h. The cells were then fixed and stained with anti-LC3B and TRITC-conjugated anti-mouse secondary antibodies for fluorescence imaging. Bar chart represents the quantitation of autophagic cells. (**F**) Inhibition of Ryanodine receptor blocked the neferine-mediated cell death in apoptosis-resistant cancer. DLD-1 Bax-Bak DKO colon cancer cells were treated with DMSO (Crtl) or neferine (10 μM) with or without ryanodine receptor inhibitor, Ryr (25 μM) for 24 h. The percentage of cell death was then assayed by annexin V staining using flow cytometry analysis. Results shown are the means ± S.D. of three independent experiments.

Since, Atg7^−/−^ MEFs are resistant to autophagy induction^6^ and neferine-induced autophagy requires Atg7 (Fig. 2D), we used both wild-type and Atg7^−/−^ MEFs to investigate whether neferine-mediated autophagy may lead to cell death^10^. Both wild-type and Atg7^−/−^ MEFs were incubated with neferine and then subjected to annexin V-PI flow cytometry analysis. Results showed that neferine significantly induced cell death in wild-type MEFs, but not in Atg7^−/−^ MEFs (Fig. 7A). Concomitantly, we further investigated the neferine-induced cell death by using an autophagy-deficient lung cancer cells H1650^33^. Results demonstrated that neferine only induced cell death in autophagy-wild type lung cancer cells H1975, but not in autophagy-deficient H1650 lung cancer cells (Supplementary Fig. S13B,C). As shown in Fig. 7B, wild-type HeLa cells were found to be more sensitive to neferine-induced cell death compared to HeLa cells with Atg7 silenced by siRNA, implicating that neferine-mediated autophagy ultimately leads to autophagy-dependent cell death.

In order to examine the potent cytotoxicity of neferine towards apoptosis-resistant cells, a panel of apoptosis-defective or apoptosis-resistant cells were utilized, such as caspase-3/-7/-8-deficient MEFs and Bax-Bak double knockout (DKO) MEFs. As shown in Fig. 7C, neferine displayed similar cytotoxicity profiles in both wild-type and caspase-3/-7/-8-deficient, or caspase-3/-7 DKO MEFs. Similar results were observed from both Bax-Bak wild-type and DKO MEFs. The resistance factor, defined as (IC_50_ value of tested compound in drug-resistant cell line) / (IC_50_ value of tested compound in drug-sensitive cell line), were calculated in cells treated with neferine, taxol, etoposide, cisplatin, doxorubicin, and staurosporine. In fact, the isogenic DLD-1 Bax-Bak DKO colon cancer cells demonstrated significant increase in drug-resistance, as indicated by the resistant factor, towards the chemotherapeutic agents, including taxol, etoposide, cisplatin, doxorubicin and staurosporine, from 1.84 to 64.2 fold, when compared to the isogenic wild-type cancer cells, whereas neferine indicated similar IC_50_ values in both Bax-Bak wild-type and DKO cancer cells (Fig. 7D). Of note, blocking of neferine-mediated autophagy by siRNA knockdown of Atg7 in this apoptosis-resistant Bax-Bak DKO DLD-1 cancer cells would suppress the cytotoxic effect of neferine (Supplementary Fig. S13D,E). This data suggests that neferine can circumvent the apoptosis-resistant phenotype of cancer *via* induction of autophagy-dependent cell death. Furthermore, neferine significantly induced red LC3-II puncta formation in DLD-1 Bax-Bak DKO colon cancer cells (Fig. 7E). However, neferine-induced red LC3-II puncta formation was suppressed by the addition of RyR inhibitor (ryanodine, Ryr) or BM, confirming that neferine induced autophagy in apoptosis-resistant cancer *via* the RyR and the Ca^2+^ signaling pathway (Fig. 7E). Concomitantly, addition of 25 μM Ryr indicated a weak basal level of Ca^2+^ signaling, whereas 15 μM of neferine demonstrated a significant change in Ca^2+^ dynamic that was completely abolished by the co-treatment with Ryr (Supplementary Fig. S6). These observations were further supported by annexin V-PI staining analysis on cell death after neferine treatment with or without the inhibitor Ryr. As shown in Fig. 7F, the addition of Ryr can significantly reduce the percentage of cell death in DLD-1 Bax-Bak DKO cancer after neferine treatment. The results further explain the autophagic activity and mechanism of neferine in apoptosis-resistant cancer *via* Ca^2+^ release from RyR. In addition, live cell imaging was adopted to unravel the progression of neferine-induced autophagy and autophagy-dependent cell death in both HeLa and DLD-1 Bax-Bak DKO apoptosis-resistant cancers (Supplementary Fig. S7 and Supplementary Videos 2 & 3).

**Figure 8:**
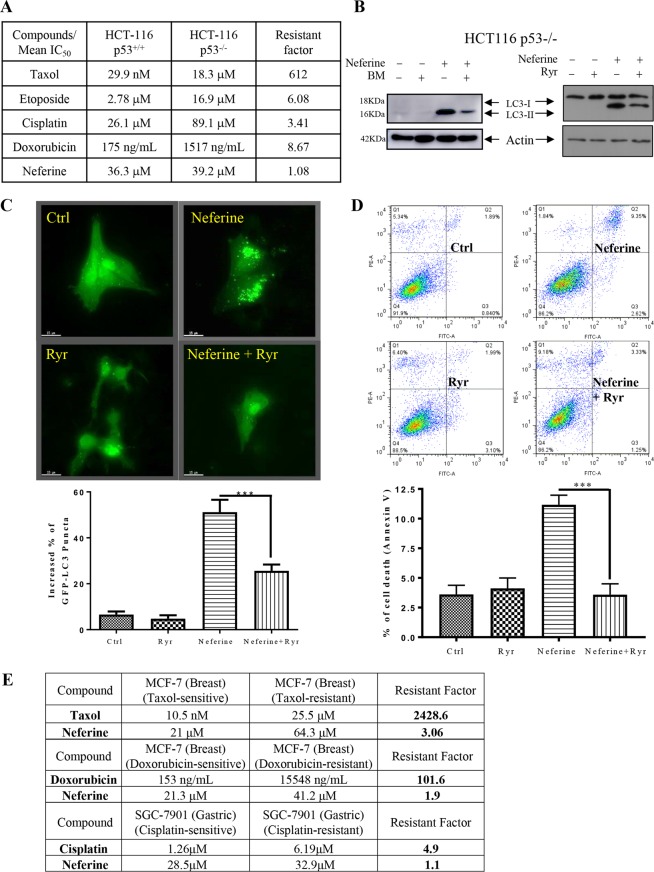
Neferine induces autophagic cell death in apoptosis-resistant HCT 116, MCF-7 or SGC-7901 cancer cells *via* activation of ryanodine receptor. (**A**) Cytotoxicity (IC_50_) of neferine and different chemotherapeutic agents towards the HCT-116 p53^+/+^ WT and apoptotic-resistant HCT-116 p53^−/−^ cell lines and drug resistance analysis (resistant factor) of the two cell lines. (**B**,**C**) Calcium chelator (BAPTA/AM, BM) and ryanodine receptor inhibitor, Ryr suppressed the neferine-induced LC3-II conversion and GFP-LC3 puncta formation in apoptosis-resistant cancer cells. The full-length blots/gels are presented in Supplementary Fig. S14. HCT-116 p53-deficient cancer cells were treated with DMSO (Ctrl), or 25 μM of neferine with or without 10 μM of calcium chelator (BAPTA/AM, BM) or 25 μM of Ryr for 24 h. Cell lysates were analyzed by western blot for LC3-II conversion (LC3-I, 18 kDa; LC3-II, 16 kDa). For immunofluorescence imaging, the drug treated HeLa cells with transiently transfection of the EGFP-LC3 were then fixed for fluorescence imaging and cells counting. Bar chart represents the quantitation of autophagic cells. (**D**) Blockage of autophagy by Ryr diminished the neferine-mediated cell death in apoptosis-resistant cancer cells. HCT-116 p53-deficient cancer cells were treated with DMSO (Crtl) or neferine (50 μM) with or without Ryr (25 μM) for 24 h. The percentage of cell death was then assayed by annexin V staining for flow cytometry analysis. (**E**) Cytotoxicity (IC_50_) of taxol, doxorubicin, and cisplatin towards the drug-sensitive and –resistant MCF-7 breast cancer cell lines as compared with neferine treatment and drug resistance analysis (resistant factor) of the two cell lines.

Furthermore, HCT-116 p53-wild-type and -deficient human colon cancer cells were also employed to validate the anti-cancer potency of neferine in apoptosis-resistant cancer (*via* p53). As shown in Fig. 8A, cytotoxicity (IC_50_) of well-known chemotherapeutic agents such as taxol, etoposide, cisplatin, and doxorubicin were found to be increased in the p53-deficient apoptosis-resistant cancer cells, whereas neferine exhibited similar cytotoxicity towards both HCT-116 p53-wild type and -deficient cancer cells, confirming the anti-cancer potency of neferine on p53 deficient apoptosis-resistant cancers. Blockage of Ca^2+^ release into the cytosol of these apoptosis-resistant cancer cells by BM or inhibition of RyR (by µM levels of Ryr) suppressed autophagy induction, as shown by the decreased LC3-II conversion (Fig. 8B) and GFP-LC3 puncta formation (Fig. 8C). Furthermore, neferine significantly induced cell death in HCT-116 p53-deficient colon cancer cells, whereas RyR blockage by Ryr also markedly abolished the neferine-mediated cell death (Fig. 8D). These data suggested that neferine-induced and autophagy-dependent cell death in p53-deficient cancer cells is mediated through Ca^2+^ release *via* RyR activation.

In addition to the comparison of the cytotoxicity of apoptotic agents to neferine by using apoptotic gene-deficient resistant cell lines, drug-mediated apoptosis-resistant cancer cells which are either sensitive or resistant to taxol, doxorubicin or cisplatin were also included for evaluation. As shown in Fig. 8E, all tested apoptotic agents including taxol, doxorubicin or cisplatin showed a high resistant factor ranging from 4.9 to 2428.6, while neferine demonstrated a resistant factor ranging from 1.1 to 3.06, indicating its similar cytotoxicity towards both drug-sensitive and -resistant cells, and further confirming its anti-cancer potency in this panel of drug-mediated apoptosis resistant cancer cells.

## Discussion

Autophagy is essential to defense against cancers, neurodegenerative disorders, ageing, and infectious diseases^34^. In light of this, small-molecules that induce autophagy may have broad therapeutic applications. Our previous studies have identified several triterpenoid- and alkaloid-based autophagic enhancers that target apoptosis-resistant cells, and have the potential of being developed into new anti-cancer agents^6,9,10^. These compounds directly inhibit SERCA to release cytosolic Ca^2+^, leading to autophagic cell death in cancer cells. In contrast, the current study demonstrated that the alkaloid compound neferine elicited similar cellular and molecular responses by activating the RyR instead of inhibiting SERCA.

Although the ability of neferine to modulate cytosolic free Ca^2+^ has been reported^35^, we are the first group correlating the autophagic effect to neferine-induced cytosolic Ca^2+^ alteration *via* RyR activation. Until recently, neferine-induced apoptosis in lung and liver cancer cells, in part *via* intracellular Ca^2+^ accumulation, have been illustrated. It has been further verified that neferine triggers autophagy activation by ROS hypergeneration and mTOR inhibition^16^. Nevertheless, pharmaceutical target(s) associated with neferine-induced and Ca^2+^-dependent autophagy induction pathway is yet to be defined. SERCA is an ER protein and considered as a potential therapeutic target for cancer therapy. For example, the well-known SERCA inhibitor TG can trigger ER stress response and apoptosis in cancer models. However, SERCA is critical to normal cellular metabolism and may produce unfavorable cytotoxicity upon inhibition^36^. Here, we firstly confirmed that neferine specifically activated SR-bound RyR (Fig. 5F,G) for Ca^2+^ release without significantly affecting SERCA or the InsP_3_ receptor. This Ca^2+^ mobilization further activated the calcium-dependent kinase^9,37^, CaMKKβ, leading to activation of the AMPK-mTOR signaling cascade (Fig. 4) and the subsequent induction of autophagy and autophagic cell death in cancer cells (Supplementary Fig. S8).

Studies using multidrug-resistant Bax-Bak deficient cells have shown that specific SERCA inhibition by TG induces necrotic cell death because of permanent mitochondrial damage resulting from Ca^2+^ overload^38^. Accordingly, the apoptosis-resistant Bax-Bak deficient cells could not be exempted from cell death, since these cells did not compromise the induction of necrosis by SERCA inhibitors^38^. Neferine possesses similar necrosis-inducing effects by upregulating cytosolic Ca^2+^ levels and inducing ER stress and mitochondrial membrane potential loss *via* activation of RyR^17,39,40^, implying that the neferine-induced cytotoxicity may simultaneously trigger autophagic cell death and necrosis in Bax-Bak deficient cells. Therefore, targeted application of ER stressors, such as neferine, represents a promising approach for treating Bax-Bak deficient or more generally drug-resistant tumors^38^.

The RyRs are a family of Ca^2+^ release channels found on intracellular Ca^2+^ storage/release organelles in many cell types, for example neurons, striated muscle cells, and smooth muscle cells, participating in a repertoire of important Ca^2+^ signaling for cellular homeostasis maintenance^41^. Recently, RyR2-mediated Ca^2+^ fluxes have been reported as the proximal controllers of mitochondrial Ca^2+^, ATP levels, and a cascade of transcription factors controlling metabolism and survival. The loss of RyR2 receptor can induce a non-apoptotic form of programmed cell death associated with increased calpain-10 but not caspase-3 activation or ER stress^42^. Concomitantly, other studies also indicated that cardiomyocyte RyRs were degraded by chaperone-mediated autophagy^43^. These findings mainly reported the loss of RyR during autophagy induction.

Taken together, our work described the potency of an autophagy-inducing compound that regulates RyR to activate Ca^2+^-mediated autophagy induction, which could serve as a novel therapeutic candidate against apoptosis-resistant cancer cells. In particular, neferine deserves further in-depth investigation, since our results demonstrate that cancer cells are more sensitive to neferine treatment. The low cytotoxicity of neferine towards normal cells, such as the cardiac cells used in this study, reinforces the clinical value of neferine in cancer therapy.

## Material and Methods

### Chemicals, plasmids, small interfering RNAs and antibodies

All compounds were purchased from Sigma unless otherwise stated. Thapsigargin (TG), compound C (CC), BAPTA/AM (BM), E64D, pepstatin A, AMD3100 and STO-609, IP3 were obtained from, Calbiochem (San Diego, CA). Neferine (>98% purity, HPLC) were purchased from the China Chengdu Biotechnology Company Ltd. (Chengdu, China). Antibodies against LC3B, p70S6 kinase, phospho-p70S6 kinase (Thr389), phospho-AMPKα (Thr172), eIF2α, phospho-eIF2α (Ser51), PERK, P-4EBP and 4EBP were purchased from Cell Signaling Technologies Inc. (Beverly, MA). ULK-1, CXCR4, p62, and RyR antibodies, and ryanodine (Ryr) were purchased from Santa Cruz Biotechnology (Santa Cruz, CA). β-actin antibodies were from Sigma (St. Louis, MO). Anti-phospho-PERK (Thr980) antibodies were from BioLegend (San Diego, CA). The ZyMax™ TRITC-conjugated anti-mouse secondary antibodies were purchased from Invitrogen (Scotland, UK). pEGFP-LC3 reporter plasmid was a.pngt from Prof. Tamotsu Yoshimori (Osaka University, Japan). siRNAs targeting Atg7, ULK-1, PERK, RyR2 or non-targeting control were obtained from Qiagen (Hilden, Germany). Primer pairs for real-time PCR of RyR isoforms:

Human-RyR1-forward: 5′-CATCAGCACGACATGAGCTT-3′

Human-RyR1-reverse: 5′-CCCACACCATGTAGCAGTTG-3′

Human-RyR2-forward: 5′-TGCAAGACTCACCGAAGATG-3′

Human-RyR2-reverse: 5′-CCACCCAGACATTAGCAGGT-3′

Human-RyR3-forward: 5′-CTGACGTCCATCTTTGAGCA-3′

Human RyR3-reverse: 5′-CAAGGGAGTAGAGGCTGCAC-3′

Rat-RyR1-forward: 5′-TCAGCAAACTGGATCGTCTG-3′

Rat-RyR1-reverse: 5′-GTGATTCCTCCCATGCTTGT-3′

Rat-RyR2-forward: 5′- TTTCGTGAGCATTAGCAACG-3′

Rat-RyR2-reverse: 5′- GAGGCACAAAGAGGAACTCG-3′

Rat-RyR3-forward: 5′- GGAAGCTAGGGGTGGTTTTC-3′

Rat RyR3-reverse: 5′- TCATATCCGGCTCATCATCA-3′

### Cell culture

All cells were obtained from the American Type Culture Collection (Rockville, MD) unless otherwise specified. Immortalized wild type and Atg7-deficient mouse embryonic fibroblasts (MEF) were kindly provided by Prof. Masaaki Komatsu (Juntendo University, School of Medicine, Japan). Immortalized wild type and Caspase 3/7-deficient MEFs were kindly provided by Prof. Richard A. Flavell (Yale University School of Medicine, United State). Immortalized wild type and Caspase 8-deficient MEFs were kindly provided by Prof. Kazuhiro Sakamaki (Kyoto University, Graduate School of Biostudies, Japan). Immortalized wild type and Bax-Bak double knockout MEFs were kindly provided by Prof. Shigeomi Shimizu (Tokyo Medical and Dental University, Medical Research Institute, Japan). HCT-116 p53 wild type and deficient human colon cancer cells were kindly provided by Prof. Vogelstein (Howard Hughes Medical Institute, the Johns Hopkins University GRCF Cell Center and Biorepository, United State). All medium supplemented with 10% fetal bovine serum (FBS) and the antibiotics (50 U/ml) penicillin, and (50 μg/ml) streptomycin (Invitrogen, Paisley, Scotland, UK). All cells were cultured in a 37 °C and 5% humidified CO_2_ incubator. All cells above the passage number of 20 were excluded from experiments.

### siRNA transfection

Atg7, ULK-1, PERK, RyR2 genes were knocked down by functionally verified FlexiTube *siRNA* (Qiagen) in HeLa cells. HeLa cells (5 × 10^5^) were seeded in six-well plate and then transfected at 70–90% confluent. 3.75 µL lipofectamine 3000 reagent (Invitrogen) and 3 µL *siRNA* [10 μM] were mixed in 250 µL Opti-MEM medium. The mixture was then incubated at room temperature for 10 min and then added dropwise into culture disc containing 1 mL medium. Transfected cells were cultured under normal culture condition (5% CO_2_, 37 °C) for 24 h and then treated with or without neferine for another 24 h. After that, the total RNA would be extracted and converted into cDNA for RT-PCR analysis, and/or the cell lysates were harvested for Western blot analysis of knockdown efficiency.

Atg7 *siRNA* target sequence: 5′-ATCAGTGGATCTAAATCTCAA-3′

ULK-1 *siRNA* target sequence: 5′-CGCGCGGTACCTCCAGAGCAA-3′

PERK (EIF2AK) *siRNA* target sequence: 5′-CACAAACTGTATAACGGTTTA-3′

RyR2 *siRNA* target sequence: 5′-CTCGTCGTATTTCTCAGACAA-3′

### Colony formation assay

300 HeLa cells, 200 H1299 cells and 500 HepG2 cells were seeded into six-well plate and incubated at 37 °C and 5% CO_2_ in the presence of 1, 2.5 and 5 μM of neferine for 2 weeks. Subsequently, the medium was removed and the cells stained with 0.1% crystal violet (Sigma, US) followed by counting positive colonies (diameter > 40 μm) after imaging. The differences in the colony formation ability of different cell types were calculated as plating efficiency (PE). PE = no. of colonies formed/ no. of cells seeded x 100%; surviving fraction (SF) = no. of colonies formed after treatment/ no. of cells seeded x PE.

### Quantification of EGFP-LC3 puncta formation

EGFP-LC3 puncta formation was quantified as described previously^10^. The EGFP-LC3 transfected cells were treated with neferine (10 μM) for indicated time and then fixed with 4% paraformaldehyde (Sigma). Coverslips were mounted onto microscope slides with FluorSave™ Reagent (Calbiochem), and localization of EGFP-LC3 was examined and captured under epifluorescence microscopy (Applied Precision DeltaVision Elite, Applied Precision, Inc, USA). Guidelines were followed to monitor autophagy^44^, the percentage of cells with punctuate EGFP-LC3 fluorescence was calculated by counting the number of the cells showing the punctuate pattern of EGFP-LC3 (≥10 dots/cell) in EGFP-positive cells over the total number of EGFP-positive cells in the same field. A minimum of 1000 cells from randomly selected fields were scored per condition per experiment.

### Cytotoxicity assays and apoptosis detection

Cell viability was measured using the MTT assay as described in^45^. The percentage of cell viability was calculated using the following formula: Cell viability (%) = Cells number_treated_ / Cells number_DMSO control_ × 100. Apoptosis was detected by Annexin V staining kit (BD Biosciences, San Diego California, USA). Flow cytometry analysis of Annexin V stained cells was carried out using a BD FACSAria III flow cytometer (BD Biosciences). Data acquisition and analysis were performed with CellQuest (BD Biosciences). Data were obtained from three independent experiments.

### Transmission electron microscopy

Cells were fixed overnight with 2.5% glutaraldehyde followed by a buffer wash. Samples were postfixed in 1% OsO4 and embedded in Araldite 502. Ultrathin sections were doubly stained with uranyl acetate and lead citrate, and analyzed using the Philips CM100 transmission electron microscope at a voltage of 80 kV.

### RT2 profiler autophagy PCR array analysis

For PCR array analysis, neferine treated HeLa cells were used to obtain the total RNA by Qiagen RNeasy® Mini Kit (Qiagen). The autophagy pathway specific RT-PCR array (Qiagen) was used to evaluate the potential alterations of related genes after neferine treatments in HeLa cells. The autophagy array comprised of 87 genes selected based on their involvement in regulating autophagy induction. There were 5 housekeeping genes served as positive controls. Total RNA was reverse-transcripted using the RT2 First Strand Kit. Real-time PCR reactions were carried out on ViiA™ 7 Real Time PCR System (Applied Biosystems) using the RT2 SYBR® Green qPCR Mastermix (Qiagen) according to manufacturer’s instructions. Data analysis was performed using the Qiagen’s integrated web-based software package for the PCR Array System, which automatically performs all ΔΔCt based fold-change calculations from raw threshold cycle data.

### Measurement of intracellular free calcium concentration

Changes in intracellular free Ca^2+^ were measured by a fluorescent dye, Fluo-3. Briefly, HeLa cells were washed twice with MEM media after neferine treatment (5 μM/10 μM) for indicated time. Cell suspensions were incubated with 5 μM Fluo-3 at 37 °Cfor 30 min. The cells were then washed twice with HBSS. The re-suspended cell samples were subjected to FACS analysis and at least 10 000 events were analyzed.

### Computational docking

The initial 3D structures for neferine were downloaded from the PubChem (http://pubchem.ncbi.nlm.nih.gov). Then, the inhibitors were preprocessed by the LigPrep^46^ which uses OPLS-2005 force field and gave the corresponding low energy conformers of the compounds. The ionized state was assigned by using Epik^47^ at a target pH value of 7.0 ± 2.0. The co-crystal structure of sarco(endo)plasmic reticulum Ca^2+^ ATPase (SERCA) complexed with TG was retrieved from the Protein Data Bank (PDB ID code 2AGV^48^). To prepare the protein for docking, the Protein Preparation Wizard module in Schrödinger 2009 was used to remove crystallographic water molecules, add hydrogen atoms, assign partial charges using the OPLS-2005 force field, assign protonation states, and minimize the structure. The minimization was terminated when the root-mean-square deviation (RMSD) reached a maximum value of 0.30 Å. In molecular docking, the prepared neferine were docked into the TG binding site of the SERCA using the Glide^49^ with the extra precision (XP) scoring mode. The docking grid box was defined using the Receptor Grid Generation tool in Glide^49^ by centering on TG in the SERCA. In molecular docking, 5000 poses were generated during the initial phase of the docking calculation, out of which best 1000 poses were chosen for energy minimization by 1000 steps of conjugate gradient minimizations. The best binding pose for neferine were considered for the further analysis.

### Measurement of SERCA activity

Purified Ca^2+^ ATPase (SERCA1A) was prepared from female rabbit hind leg muscle^50^. ATPase activity was determined using the enzyme-coupled method utilizing pyruvate kinase and lactate dehydrogenase as previously described in^51^. All SERCA inhibition data were fitted to the allosteric dose versus effect equation using Fig P (Biosoft, Cambridge, UK): Activity = minimum activity + (maximum activity − minimum activity)/(1 + ([I]/IC_50_)^P^).

### Measurement of RyR and InsP3 receptor activity

Rabbit skeletal muscle heavy SR was prepared as described in Saito *et al*.^52^. Brain microsomes were prepared as described in Mezna & Michelangeli^53^. Ca^2+^ release experiments from SR and brain microsomes were performed using Fluo-3 free acid as described in Tovey *et al*.^32^. Briefly, rabbit fast-twitch skeletal muscle SR (50 µg) or rat brain microsomes (500 µg) were suspended in 2 mL of a buffer containing; 60 mM KCl, 40 mM potassium phosphate, 3.5 mM potassium pyrophosphate, 0.25 µM Fluo-3 (free acid), 10 µg/ml creatine kinase and 10 mM phosphocreatine, pH 7.2, into a stirred fluorescence cuvette in a spectrofluorimeter. Ca^2+^ uptake was then initiated by the addition of 1.5 mM Mg-ATP and monitored by measuring Emission at 526 nm, while exciting at 506 nm. Once sufficient uptake had taken place neferine or IP_3_ was added.

### Measurement of cytoplasmic calcium dynamic

Intracellular cytosolic Ca^2+^ dynamic was measured using the FLIPR Calcium 6 Assay Kit (Molecular Devices) according to the manufacturer’s instructions. In brief, HeLa cells were plated at a density of 10 000 cells per well in black wall/clear bottom 96-multiwell plates from Costar (Tewksbury, MA, USA) and cultured for 24 h before treatment. After that, calcium 6 reagent was added directly to cells, and cells were incubated for an additional 2 h in the absence of external Ca^2+^ in HBSS buffer at 37 °C and 5% CO_2_. 10 and 20 μM of neferine were then added to the wells and immediately subjected to data acquisition on the FLIPR Tetra High-Throughput Cellular Screening System (Molecular Devices) at room temperature using a 1- s reading interval throughout the experiments.

### Single cell calcium imaging

2 × 10^5^ HeLa cells were cultured in 35 mm confocal disc at 37 °C CO_2_ incubator for 24 h. 5 mM of Fluo 3/AM/DMSO stock solution was diluted to 5 μM working solution using Ca^2+^ free Hanks-balanced salt solution (HBSS) and then added to cells at 37 °C for 30 min. HeLa cells were then washed 3 times with HEPES buffer saline and incubated at 37 °C in an imaging chamber for another 10 min. Changes in cytosolic [Ca^2+^] levels were monitored by following changes in fluo-3 fluorescence upon addition of 10 μM neferine in Ca^2+^ free HBSS buffer, using the real-time mode for 5 minutes by epifluorescence microscopy (Applied Precision DeltaVision Elite, Applied Precision, Inc, USA). Data Inspection Program provided by the DeltaVision software was used to measure the intensity of the fluo-3 fluorescence and the mean fluorescence intensity was monitored at 523 nm and plotted against time (sec).

### Live-cell imaging

Autophagy induction was visualized in HeLa cells or Bax-Bak^−/−^ MEFs which were transiently transfected with EGFP-LC3 by LTX transfection kit (Invitrogen), and then placed on the microscope stage covered with a 37 °C chamber in which a humidified premixed gas consisting of 5% CO_2_ and 95% air was infused. After treatment with neferine (10 μM), the cells were observed using 60X Olympus PlanApoN 1.42 oil objective, and the fluorescence monitored at 512 nm. Both DIC and fluorescent images were acquired at 5-min intervals using high magnification wide field epifluorescence microscopy. Images were captured as serial Z-sections of 1.0 μm interval by a Photometrics CoolSNAP HQ^2^ CCD camera on the Olympus IX71-Applied Precision DeltaVision restoration microscope (Applied Precision, Inc, USA), and the epifluorescence images were numerically deconvolved using DeltaVision algorithms (Applied Precision, Inc.).

### Statistical analysis

The results were expressed as means ± SD as indicated. The differences were considered statistically significant when the *P*-value was less than 0.05. Student’s *t*-test or one-way ANOVA analysis was used for comparison among different groups.

## Supplementary information

Supplementary video-1.

Supplementary video-2.

Supplementary video-3.

Supplementary figures 1–14.
