# Supplementary material for: Neferine induces autophagy-dependent cell death in apoptosis-resistant cancers via ryanodine receptor and Ca2+-dependent mechanism
Source: Sci Rep. 2019 Dec 27;9:20034. doi: 10.1038/s41598-019-56675-6 (PMC6934498; doi:10.1038/s41598-019-56675-6)
Supplement: Supplementary file 4 — Supplementary figures 1–14. [file 41598_2019_56675_MOESM4_ESM.pdf]

Neferine induces autophagy-dependent cell death in apoptosis-resistant cancers *via* ryanodine receptor and Ca^{2+}-dependent mechanism

Betty Yuen Kwan Law[a], Francesco Michelangeli[b,c], Yuan Qing Qu[a], Su-Wei Xu[d], Yu Han[a], Simon Wing Fai Mok[a], Ivo Ricardo de Seabra Rodrigues Dias[a], Masood-ul-Hassan Javed[e], Wai-Kit Chan[a], Wei-Wei Xue[f], Xiao-Jun Yao[a,f], Wu Zeng[a], Hui Zhang[a], Jing-Rong Wang[a], Liang Liu[a*] and Vincent Kam Wai Wong[a*]

[a] State Key Laboratory of Quality Research in Chinese Medicine, Macau University of Science and Technology, Macau, China
[b] School of Biosciences, University of Birmingham, Edgbaston, Birmingham, UK
[c] Department of Biological Sciences, University of Chester, Chester, UK
[d] Department of Basic Medicine of Zhuhai health school, Zhuhai, China
[e] College of Medicine, King Saud Bin Abdulaziz University of Health Sciences, King Abdul Aziz Medical City, Jeddah 21423, Kingdom of Saudi Arabia
[f] College of Chemistry and Chemical Engineering, Lanzhou University, Lanzhou, Gansu 730000, P. R. China

Running title: Neferine induces autophagic cell death *via* RyR

***Corresponding authors:** Dr. Vincent Kam Wai Wong and Prof. Liang Liu

Address correspondence: State Key Laboratory of Quality Research in Chinese Medicine, Macau University of Science and Technology, Avenida Wai Long, Taipa, Macau, China
Tel.: +853-8897-2408 (VKWW); +853-8897-2238 (LL);
Fax: +853-2882-2799 (VKWW); +853-2882-3312 (LL);
E-mail address: bowaiwong@gmail.com (VKWW); lliu@must.edu.mo (LL)

Supplementary Figure
(MTT graphs of cell lines in Figure 1B)

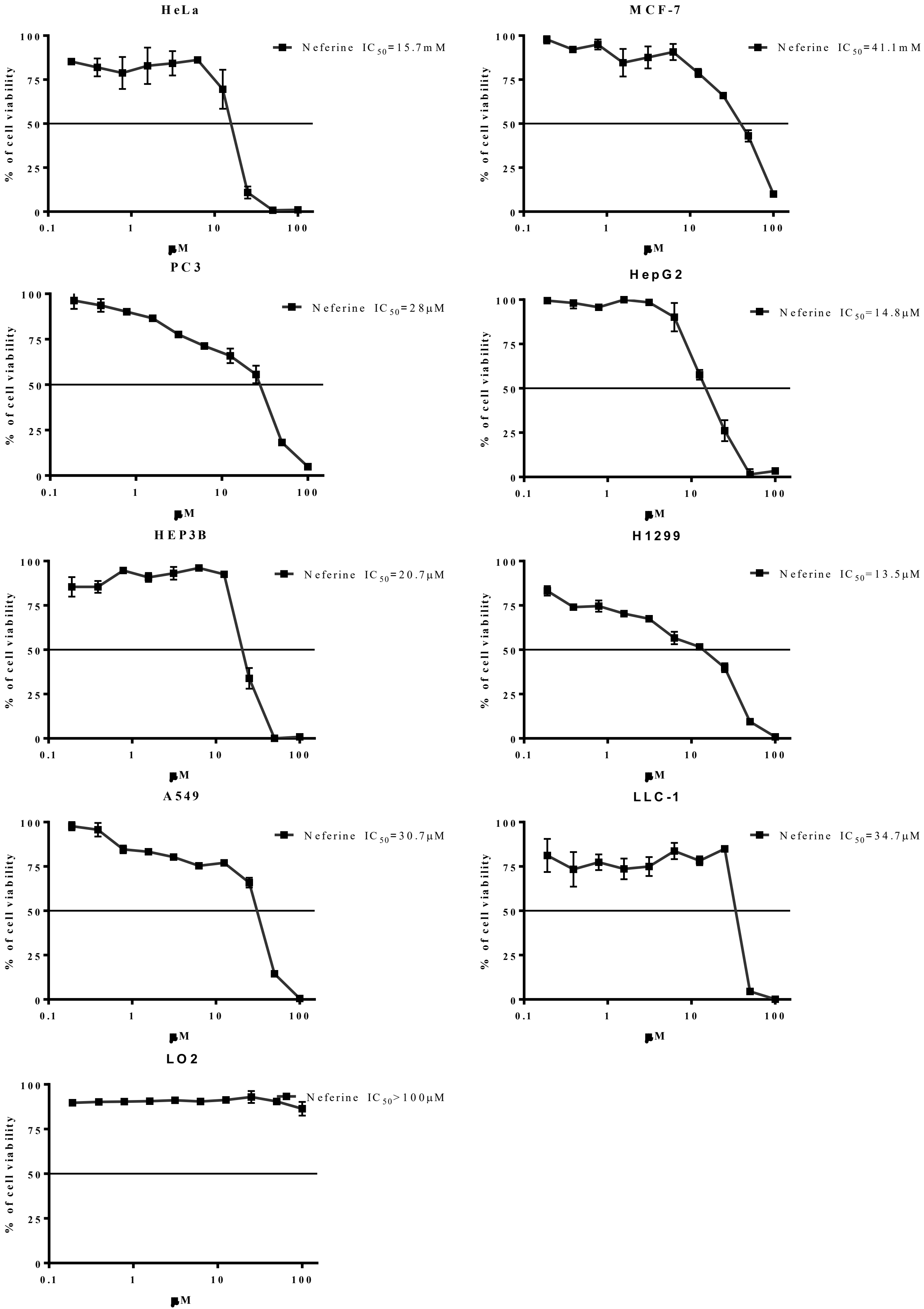

Supplementary Figure
(Quantification graphs of Figure 2A & 3B)

Figure 2A

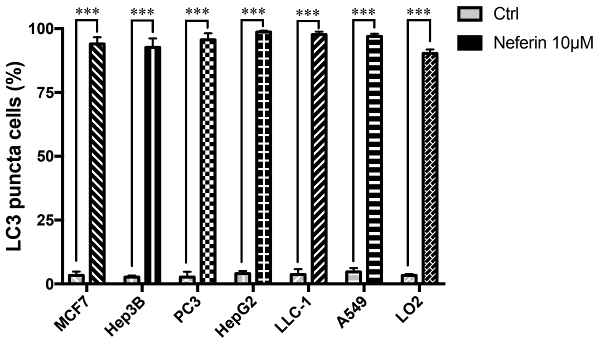

Figure 3B CXCR4

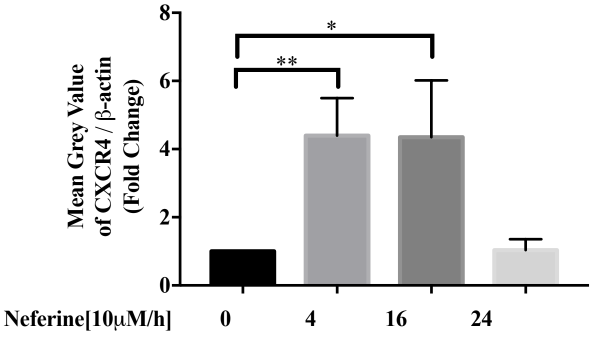

Figure 3B P-PERK/PERK

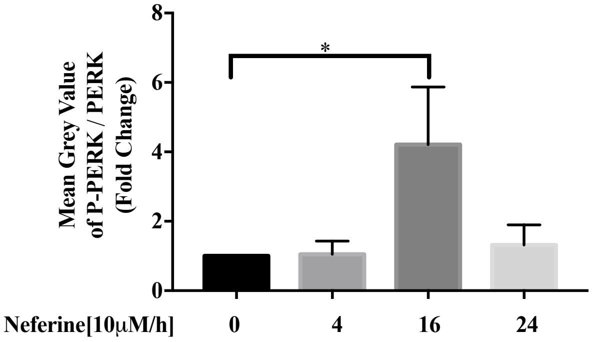

Figure 3B P62

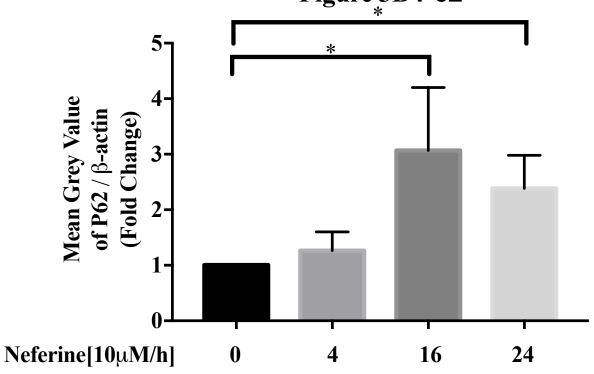

Figure 3B ULK-1

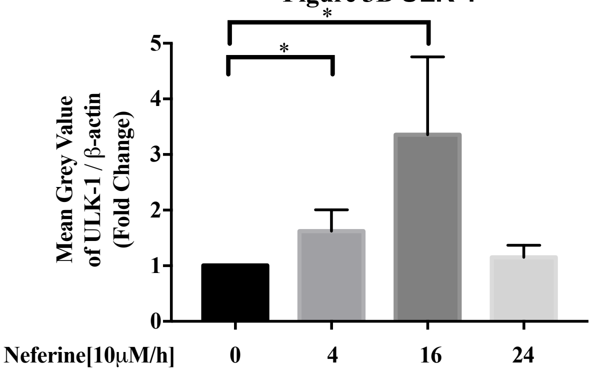

Figure 3B P-eIF-2α/eIF-2α

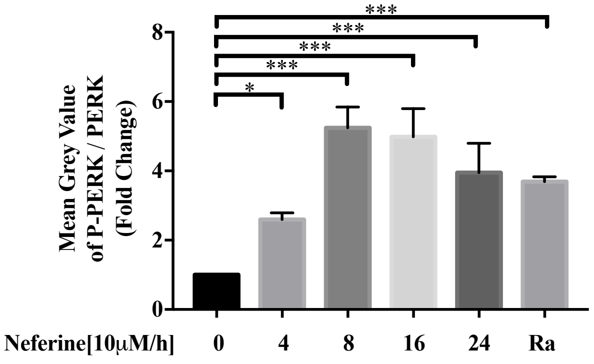

Supplementary Figure S2. The quantification of green endogenous LC3-II puncta formation (%) in Fig. 2A, compared to control group, $***P \leq 0.001$, *t test* analysis. And the quantification of mean grey value of western blot in Fig. 3B, compared to control group, $*P < 0.05$, $**P \leq 0.01$, $***P \leq 0.001$, one-way ANOVA analysis.

Supplementary Figure
(Quantification graphs of Figure 4A & 4C)

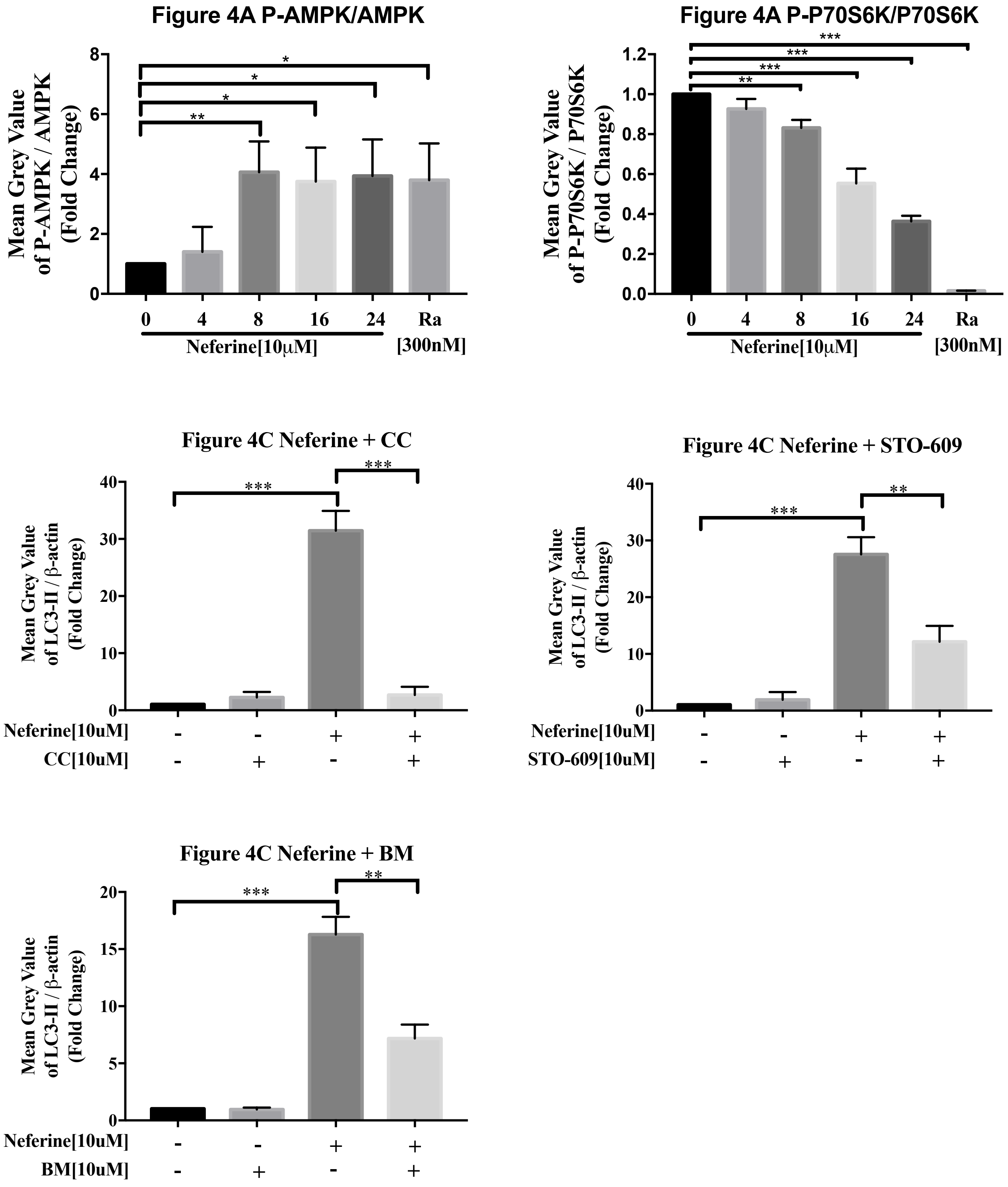

Supplementary Figure S3. The quantification of mean grey value of western blot in Fig. 4A, compared to control group, $*P < 0.05$, $**P \leq 0.01$, $***P \leq 0.001$, one-way ANOVA analysis. And the quantification of mean grey value of western blot in Fig. 4C, compared to neferine [10μM] group, $**P \leq 0.01$, $***P \leq 0.001$, one-way ANOVA analysis.

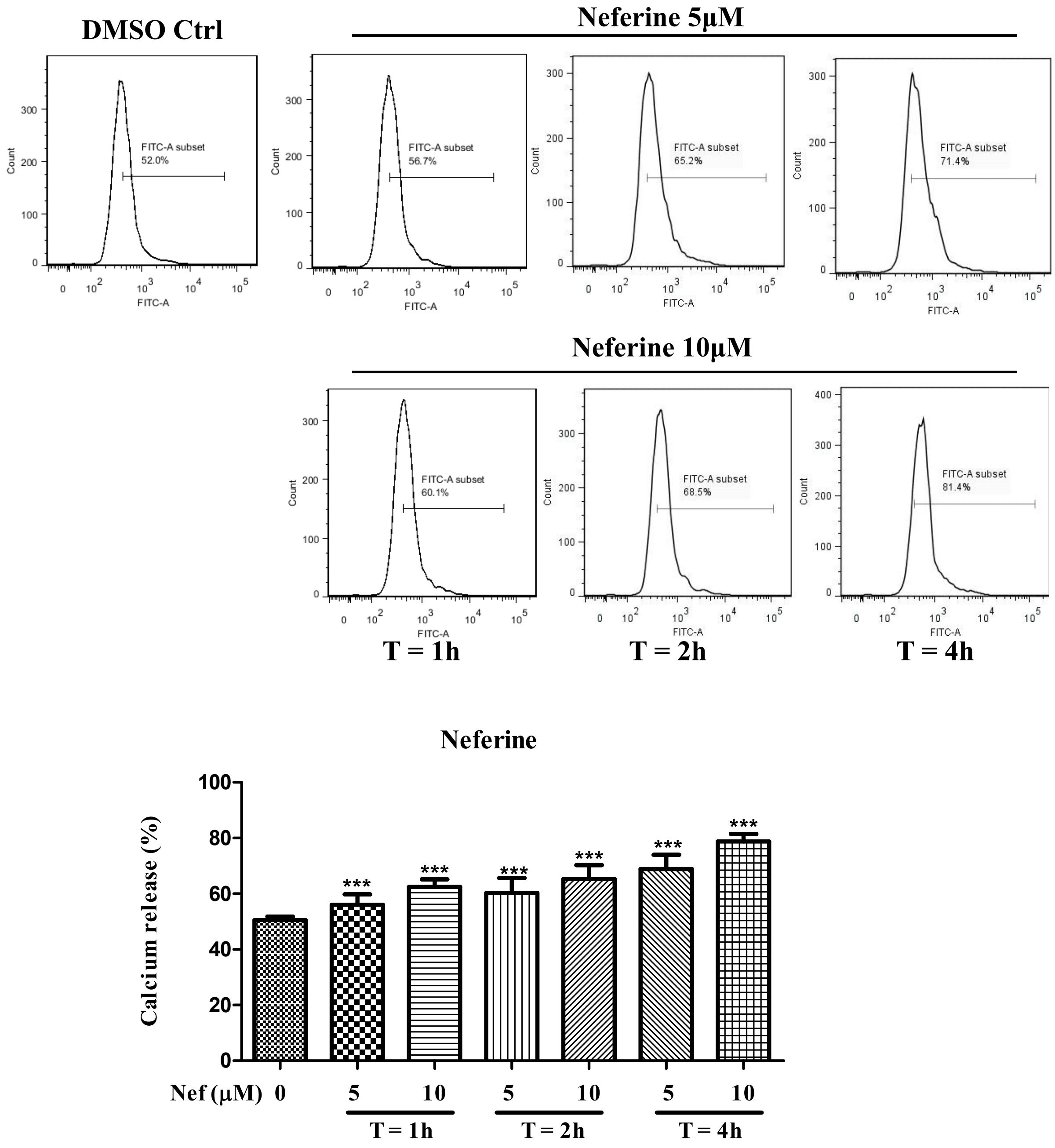

Supplementary Figure S4. Nef mobilized cytosolic calcium level in dose and time dependent manner. HeLa cells were treated with 5 or 10 µM of Nef for indicated time. Intracellular Ca^{2+} concentrations were determined in terms of the fluorescent activity of Fluo-3 by FACS analysis. FACS charts shown are the representative of three independent experiments. Bar charts indicate the percentage of calcium release in cells; error bars, S.D. ***, $P < 0.001$.

Supplementary Figure
(RyR1/2/3 genes expression level in Figure 1B cell lines)

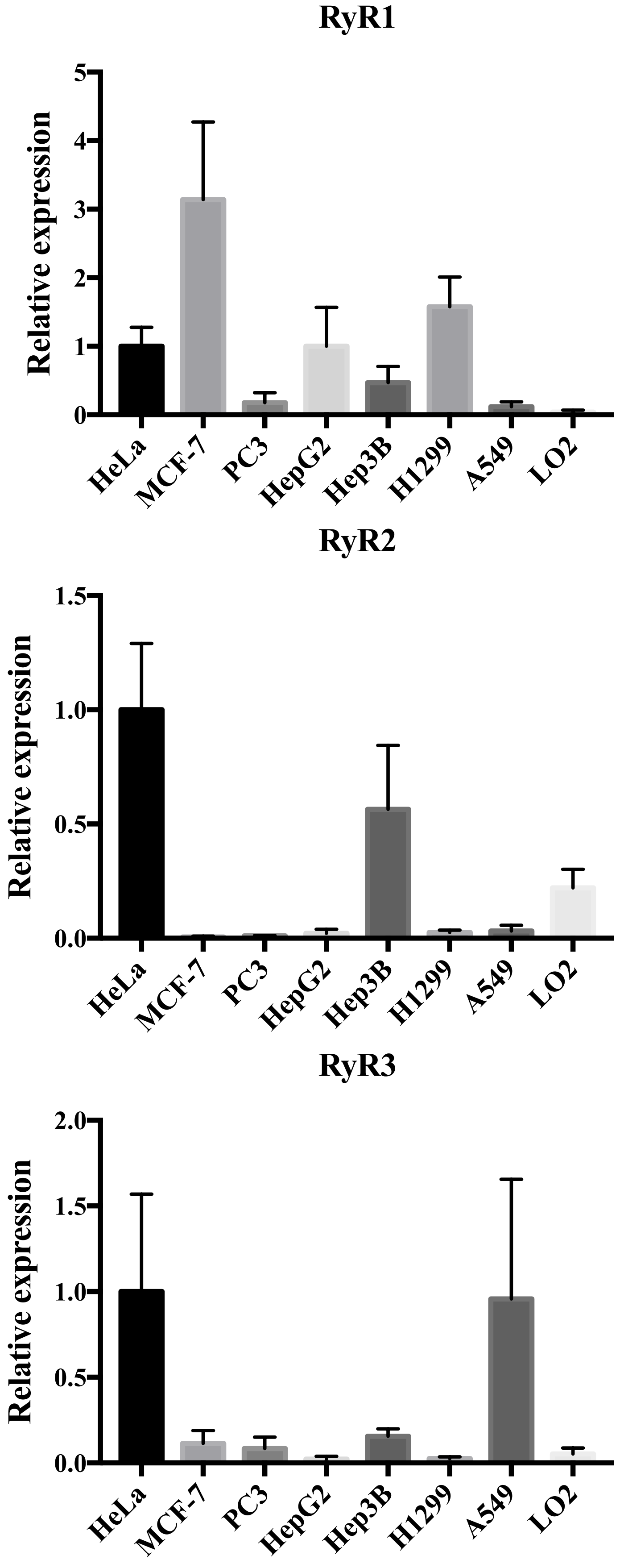

Supplementary Figure S5. The relative expression level (normalized to HeLa) of RyR 1/2/3 genes in 8 cell lines preformed in Fig. 1B.

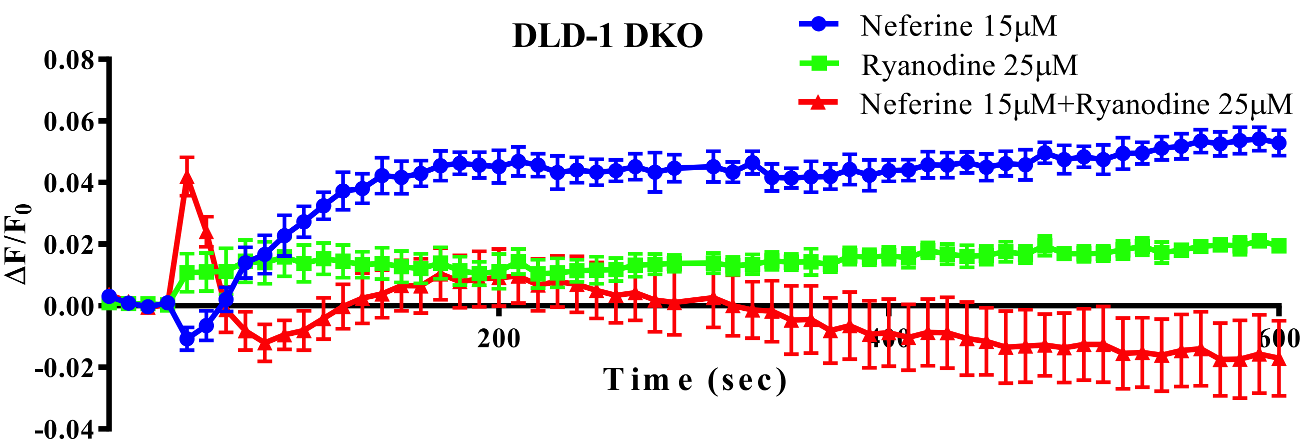

Supplementary Figure S6. Neferine-induced calcium dynamic change in DLD-1 Bax-Bak DKO colon cancer cells were abolished by addition of ryanodine receptor inhibitor (Ryanodine). DLD-1 DKO cancer cells stained with FLIPR Calcium 6 Assay Kit were treated with 15 μM of neferine in the presence or absence of 25 μM ryanodine, then immediately subjected to calcium dynamic measurement by FLIPR Tetra High-Throughput Cellular Screening System. Data from the chart represents mean values ± S.D. of three independent experiments.

Supplementary Figure S7

A

HeLa

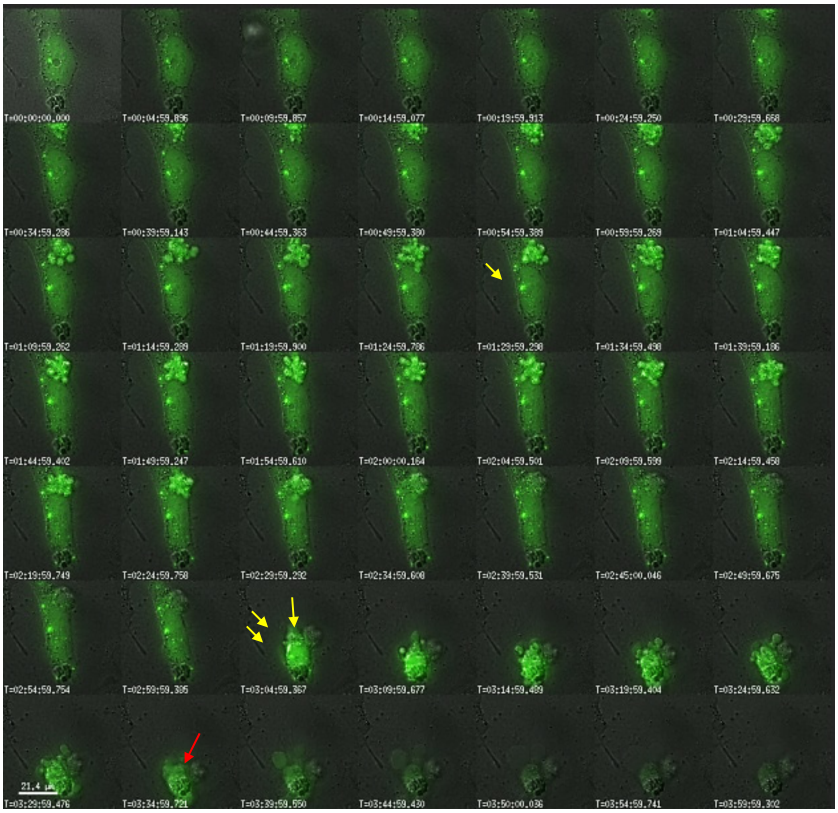

B

DLD1 Bax-Bak-DKO

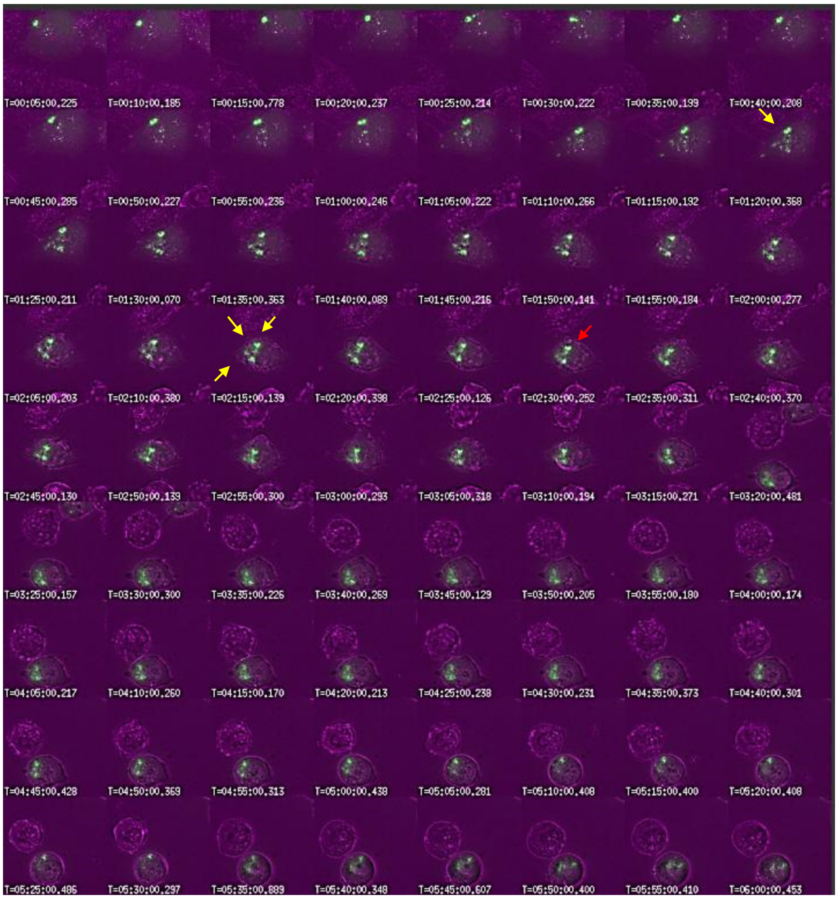

Supplementary Figure S7. Live-cell imaging of Nef-mediated autophagic cell death. EGFP-LC3 transfected HeLa cancer cells or apoptosis-resistant cells, DLD1 Bax-Bak DKO cancer cells were incubated at imaging chamber with 37℃ supplied with 5% of CO_2. After addition of 10 μM of neferine, live-cell imaging was started to record the fluorescence and bright field images for 4 h with 10 min time-intervals. Arrows (yellow), autophagy induction with EGFP-LC3 accumulation (green); arrows (red), autophagic cell death.

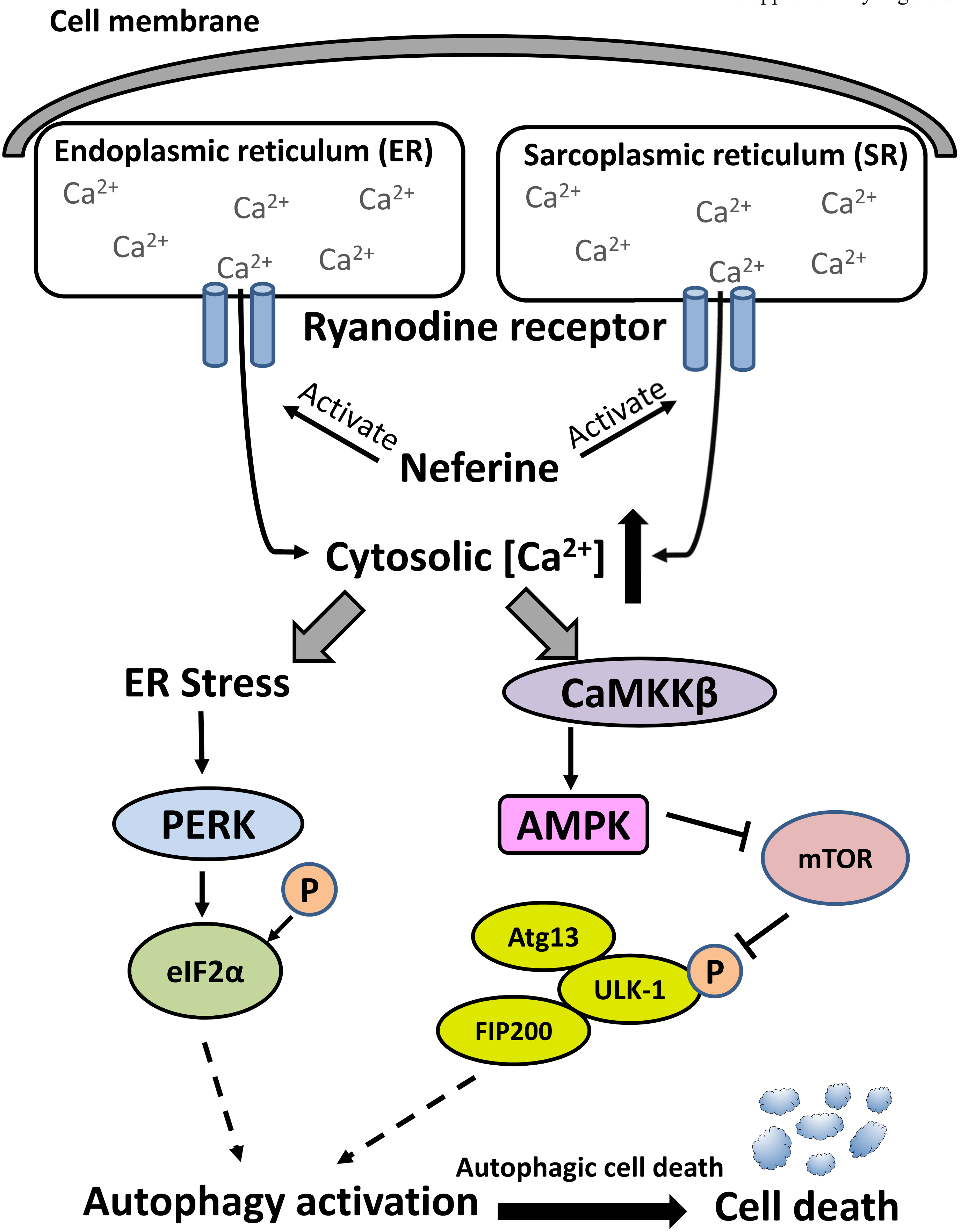

Supplementary Figure S8. Schematic diagram to illustrate the proposed molecular target and mechanisms of neferine in autophagy induction and its subsequent autophagic cell death.

Supplementary Figure
(Uncropped western blot images)

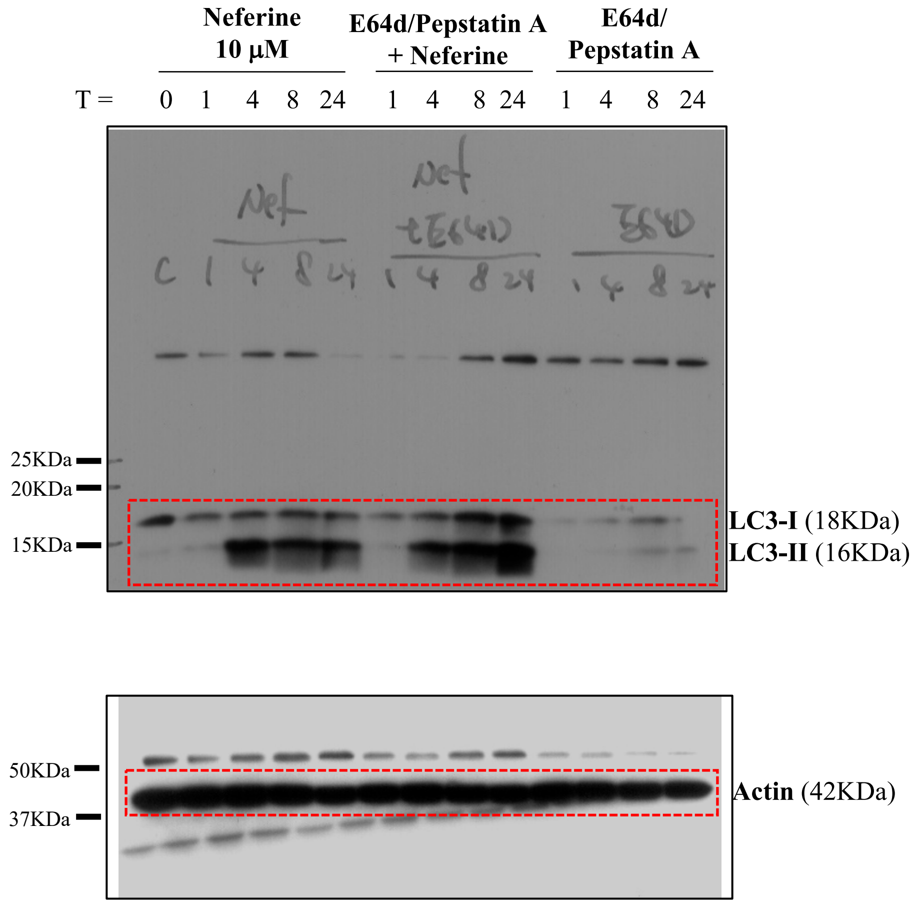

Uncropped western blot images of Figure 2C

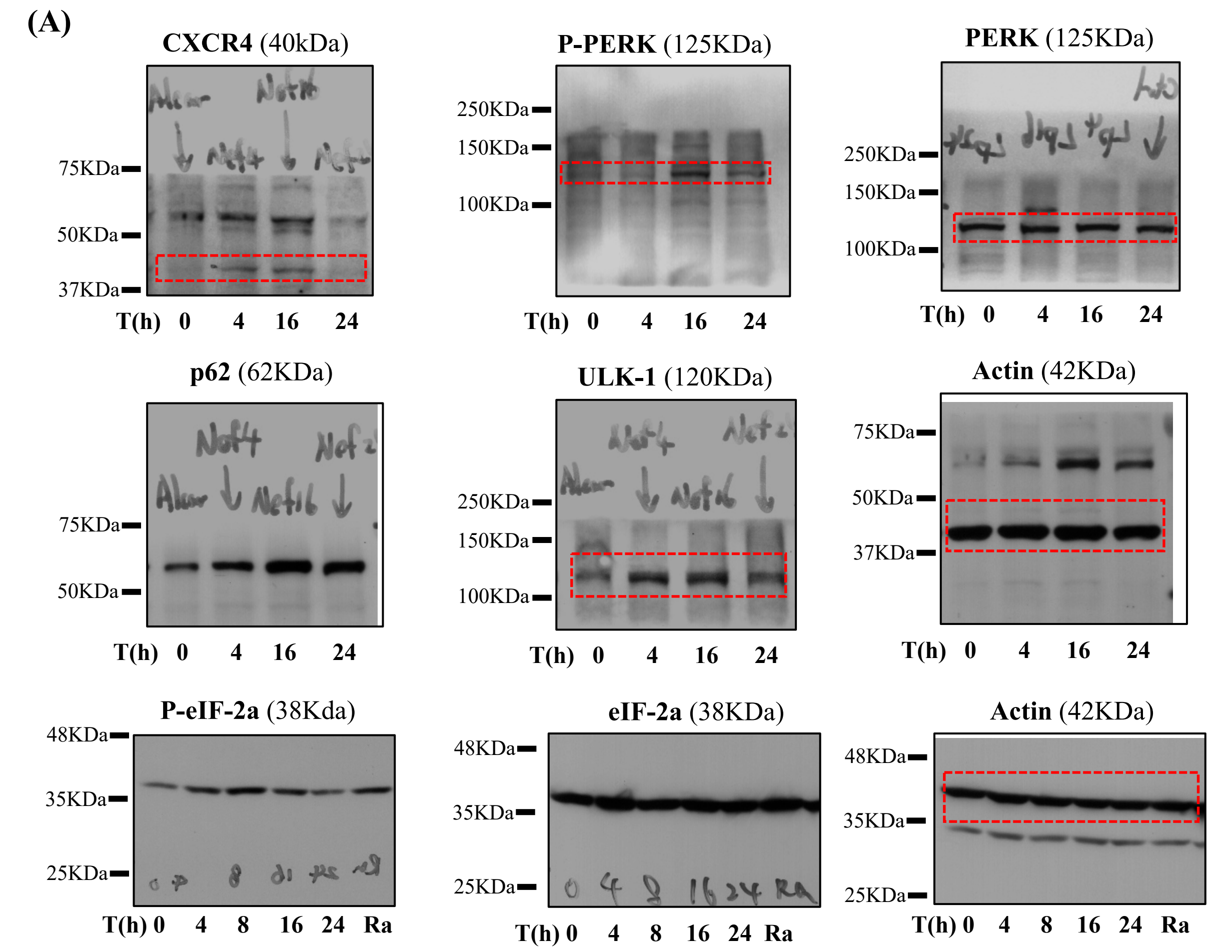

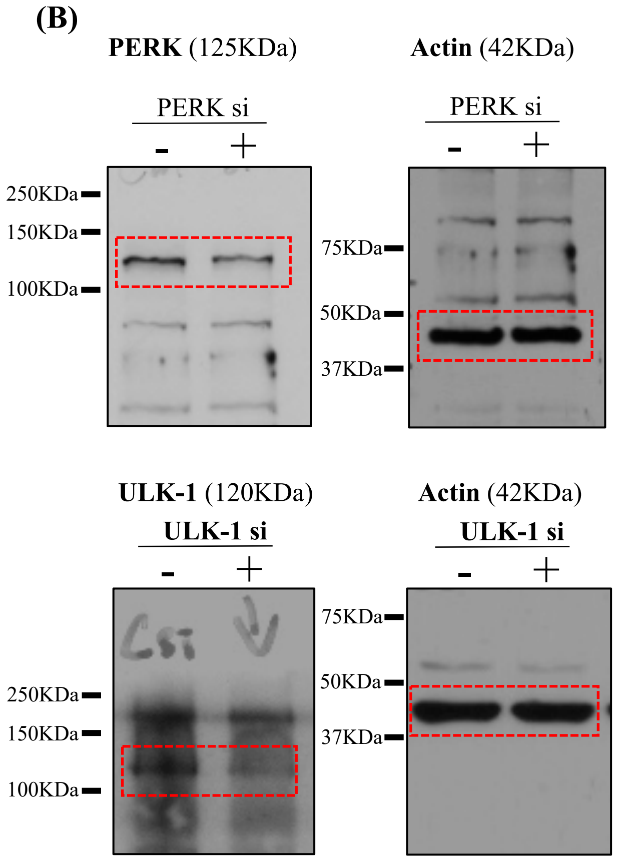

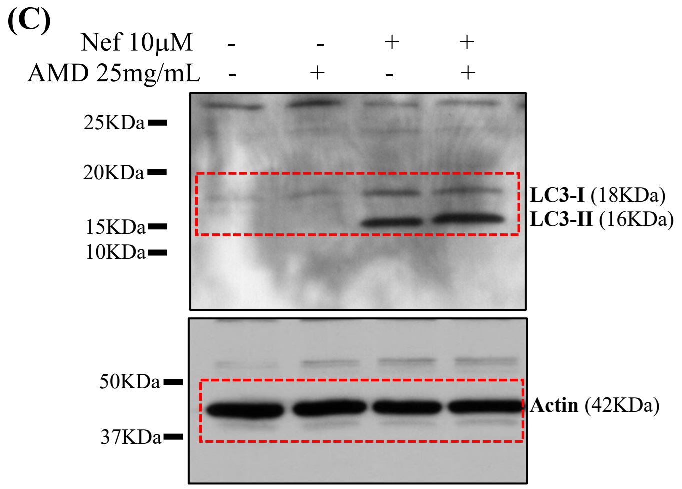

(A) Uncropped western blot images of Figure 3B. (B) Uncropped western blot images of Figure 3C. (C) Uncropped western blot images of Figure 3D .

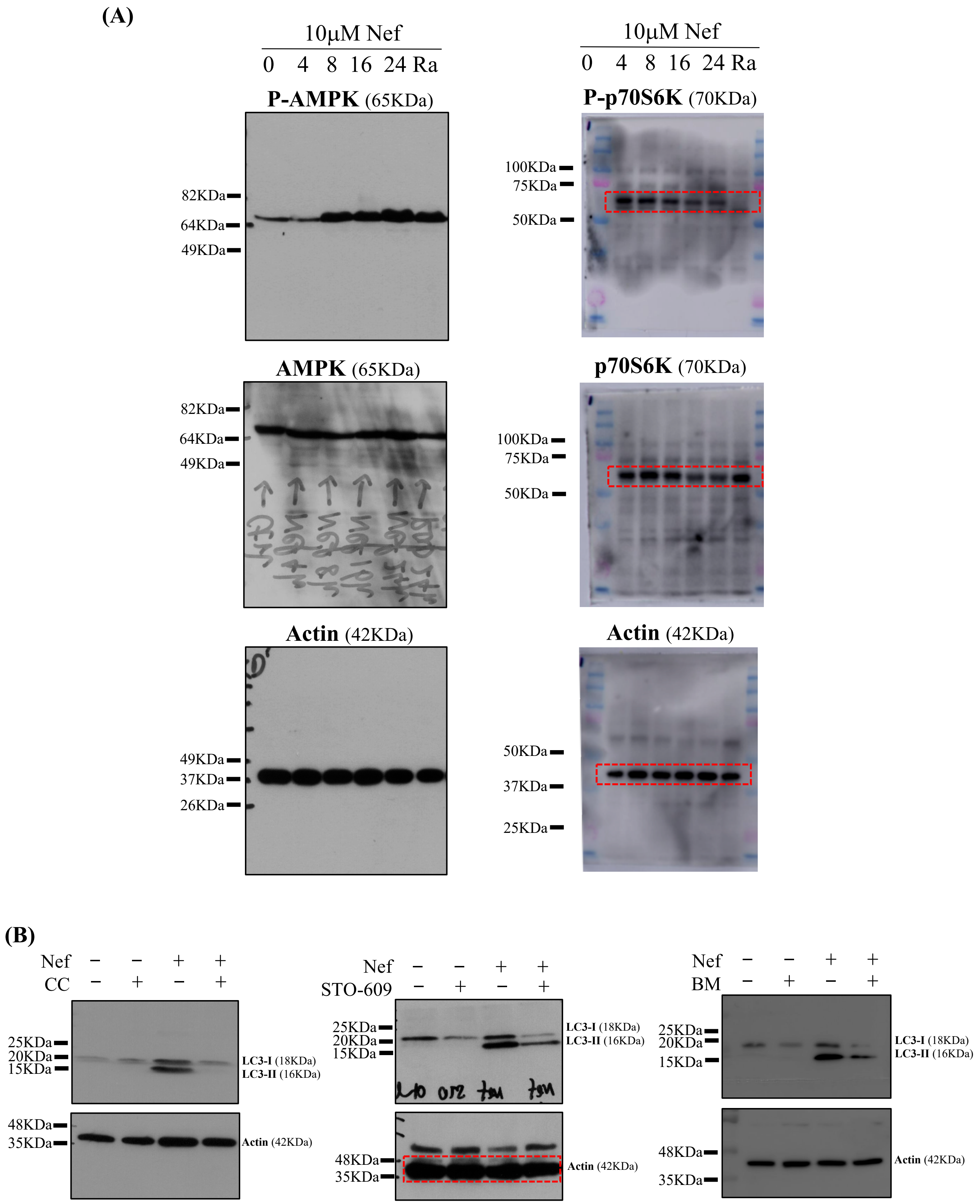

(A) Uncropped western blot images of Figure 4A. (B) Uncropped western blot images of Figure 4C.

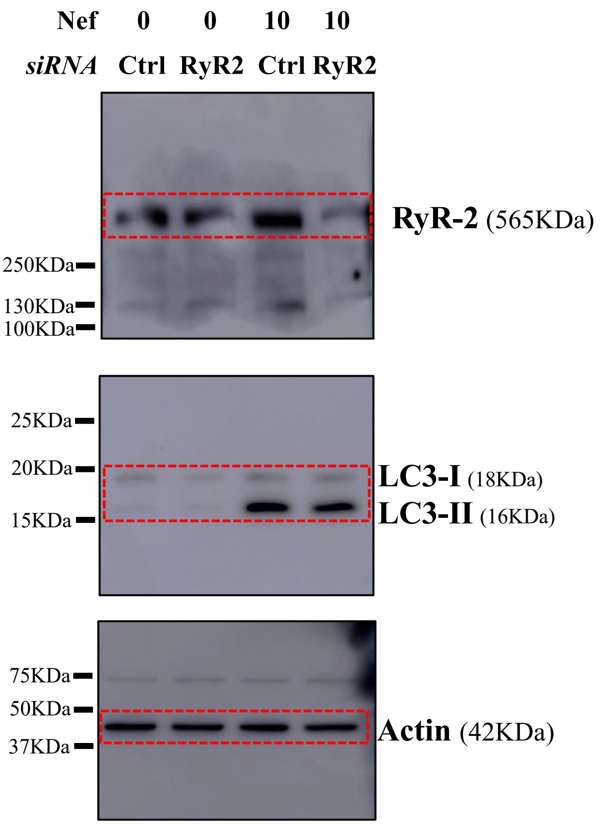

Uncropped western blot images of Figure 6B

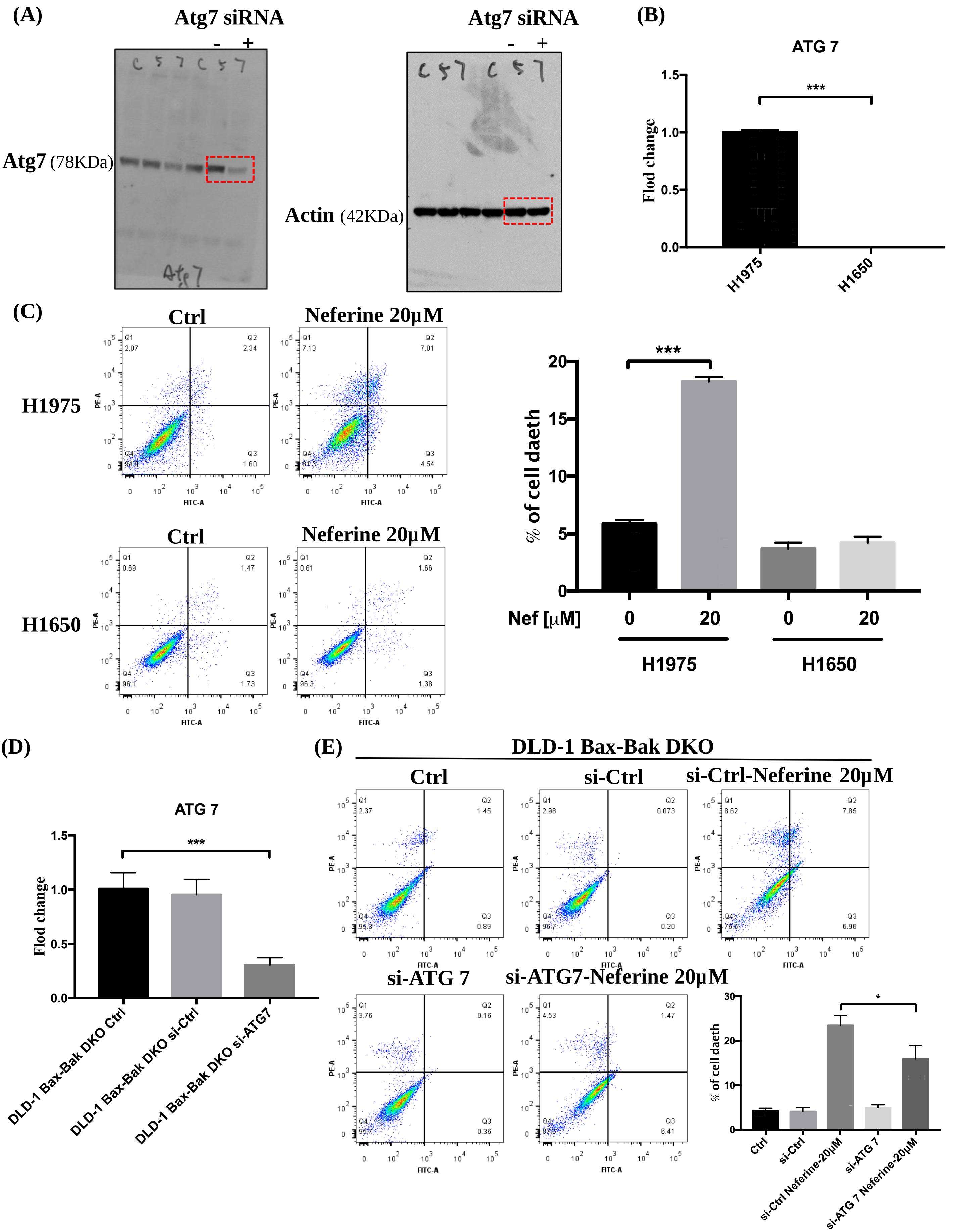

(A) Uncropped western blot images of Figure 7B. (B) Expression level of ATG7 gene in H1975 and H1650 cells. (C) Cell death flow cytometry analysis of neferine on Atg7 wild-type cells (H1975) and deficient cells (H1650). (D) The expression levels of the ATG7 gene knocked down by ATG7-siRNA in DLD-1 Bax-Bak DKO cells. (E) Cell death flow cytometry analysis of neferine on DLD-1 Bax-Bak DKO ATG7 wild-type cells and ATG7 knocked down cells.

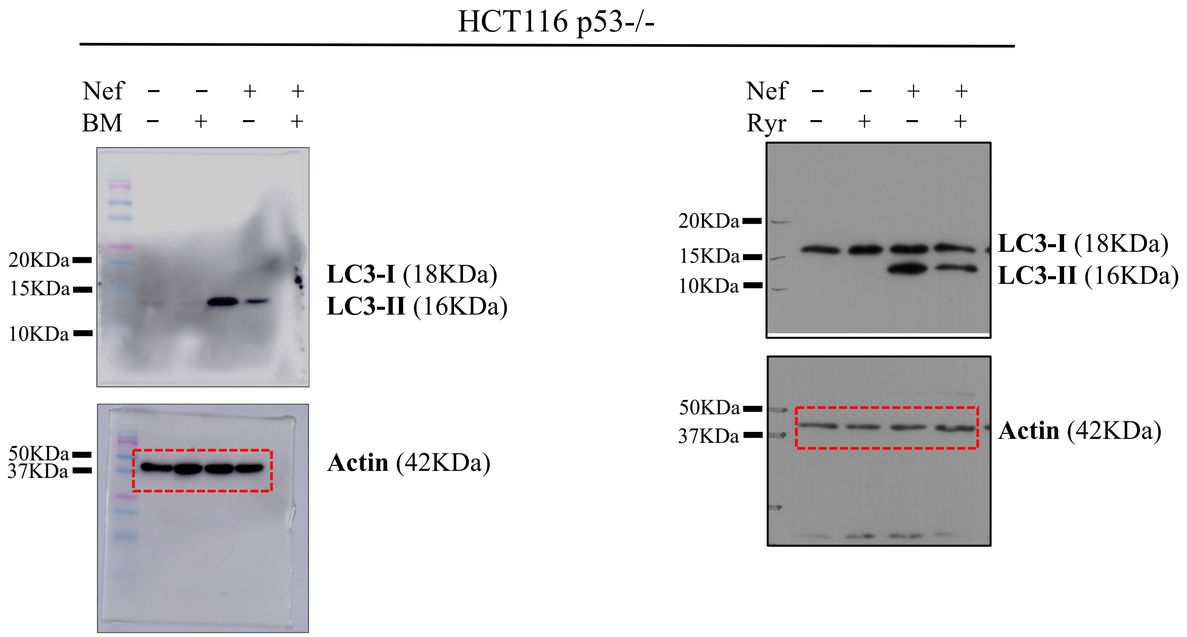

Uncropped western blot images of Figure 8B.
